# Toward Autonomous UAV Swarm Navigation: A Review of Trajectory Design Paradigms

**DOI:** 10.3390/s25185877

**Published:** 2025-09-19

**Authors:** Kaleem Arshid, Ali Krayani, Lucio Marcenaro, David Martin Gomez, Carlo Regazzoni

**Affiliations:** 1 Department of Engineering and Naval Architecture (DITEN), University of Genoa, 16145 Genoa, Italy; ali.krayani@ieee.org (A.K.);; 2 Intelligent Systems Lab (LSI), Department of Systems Engineering and Automation, Carlos III University of Madrid, 28911 Leganés, Spain; dmgomez@ing.uc3m.es

**Keywords:** unmanned aerial vehicles, UAV swarm, trajectory planning, collision avoidance, MTSP, multi-agent systems, artificial intelligence, bio-inspired algorithms, swarm robotics

## Abstract

The development of efficient and reliable trajectory-planning strategies for swarms of unmanned aerial vehicles (UAVs) is an increasingly important area of research, with applications in surveillance, search and rescue, smart agriculture, defence operations, and communication networks. This article provides a comprehensive and critical review of the various techniques available for UAV swarm trajectory planning, which can be broadly categorised into three main groups: traditional algorithms, biologically inspired metaheuristics, and modern artificial intelligence (AI)-based methods. The study examines cutting-edge research, comparing key aspects of trajectory planning, including computational efficiency, scalability, inter-UAV coordination, energy consumption, and robustness in uncertain environments. The strengths and weaknesses of these algorithms are discussed in detail, particularly in the context of collision avoidance, adaptive decision making, and the balance between centralised and decentralised control. Additionally, the review highlights hybrid frameworks that combine the global optimisation power of bio-inspired algorithms with the real-time adaptability of AI-based approaches, aiming to achieve an effective exploration–exploitation trade-off in multi-agent environments. Lastly, the article addresses the major challenges in UAV swarm trajectory planning, including multidimensional trajectory spaces, nonlinear dynamics, and real-time adaptation. It also identifies promising directions for future research. This study serves as a valuable resource for researchers, engineers, and system designers working to develop UAV swarms for real-world, integrated, intelligent, and autonomous missions.

## 1. Introduction

Unmanned aerial vehicles (UAVs) have emerged as a revolutionary technology in defence, commercial, and scientific fields over the past decade. In particular, UAV swarms have played a significant role in areas such as intelligence, surveillance, search and rescue, agricultural inspection, natural disaster monitoring, and communication networks [1,2]. The scalability of UAV swarms makes them suitable for complex, collaborative missions where multiple UAVs must operate simultaneously and effectively in dynamic and uncertain environments.

The basic requirement for the safe and efficient operation of UAV swarms is that each UAV not only plans its trajectory autonomously but also flies in coordination with other UAVs to avoid collisions, resource wastage, and communication bottlenecks [3,4]. This coordination can depend on both centralised and decentralised control architectures. Centralised systems rely on a central controller that manages the planning of all UAVs, while decentralised systems have each UAV relying on local information and communicating with neighbouring UAVs. Understanding this distinction is crucial for planning the trajectories of UAV swarms.

Moreover, it is essential to differentiate between trajectory planning/design and path planning: path planning primarily focuses on finding the shortest route, while trajectory planning incorporates time, velocity, acceleration, and the physical constraints of the UAV [5,6]. Trajectory planning in swarm missions is often modelled as a Multiple Travelling Salesman Problem (MTSP), where multiple UAVs must cover different targets while considering mission time, energy constraints, and inter-UAV safety. To address these challenges, the research community has proposed various approaches to trajectory planning. Three major paradigms stand out:

Traditional Algorithms (TA): Deterministic methods such as Dijkstra [7], A [8], and Dubins Curves [9], which rely on complete environmental information and provide optimal or near-optimal paths in well-structured scenarios [10,11,12].

Biologically Inspired Algorithms (BIA): Approaches inspired by natural phenomena, such as bird flocking or the pheromone trails of ants, including PSO [13], ACO [14], GA [15], and ABC [16], which provide global optimization in large and complex search spaces [17].

Modern AI-based Algorithms (AI-A): Machine learning [18], deep learning [19], reinforcement learning (RL) [20], multi-agent RL (MARL) [21], and graph neural networks enable UAV swarms to perform adaptive decision making, collaborative coordination, and intelligent behaviour in dynamic, uncertain environments [22,23]. In particular, modern approaches such as Active Inference [24], based on Bayesian foundations, are introducing new directions in trajectory planning through predictive processing [25].

These approaches are interconnected and form a continuum. TAs provide a foundational structure, BIAs offer global exploration and diversity, and AI-based techniques enable real-time adaptability and intelligent decision making. In modern research, these methods are being integrated into hybrid frameworks to simultaneously address complex aspects of trajectory design, such as scalability, collision avoidance, and mission-level optimisation.

The main objective of this paper is to present a systematic, comprehensive, and analytical review of all the essential aspects of UAV swarm trajectory planning, highlighting the clear connections and differences between various approaches.

- This study outlines the fundamental concepts of centralised and decentralised control architectures and their practical applications.
- The fundamental difference between trajectory design and path planning is clarified, and MTSP is introduced as a central mathematical framework that has been effectively adopted in UAV swarm trajectory planning.
- The study discusses online and offline training/testing approaches, detailing how AI-based methods can be trained using an offline-generated BIA-based dataset and subsequently enhanced through online testing and minor adaptations in real-world missions.
- The study clarifies decision making and collision avoidance as core challenges of UAV swarm trajectory planning and analyses various scientific approaches to solving these problems using geometric, physics-based, and AI-driven techniques.
- This investigation provides a comparative analysis and critically evaluates the strengths and limitations of each approach, ultimately outlining future directions for UAV swarm research.

**Figure 1:**
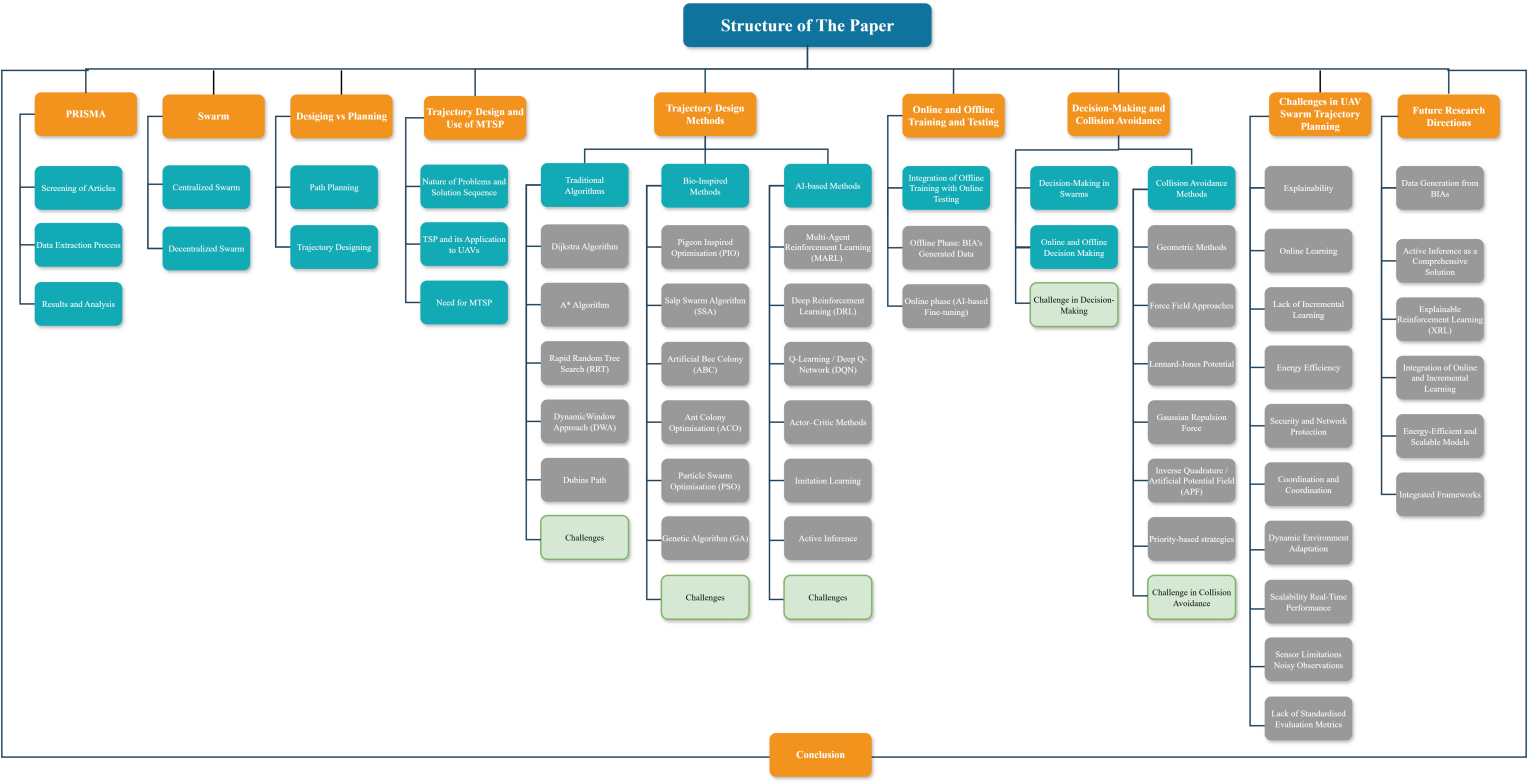
Structure of the paper.

The structure of the paper is depicted in Figure 1 and is organised as follows: First, centralised and decentralised swarm approaches are discussed, followed by the distinction between trajectory design and path planning. Next, the MTSP problem and its application to UAV swarms are described. TA, BIAs, and modern AI-based strategies are then presented. Subsequently, aspects of online and offline training/testing, decision making, and collision avoidance are reviewed. Ultimately, the paper highlights the primary challenges and potential future directions of UAV swarms.

## 2. Method

**Figure 2:**
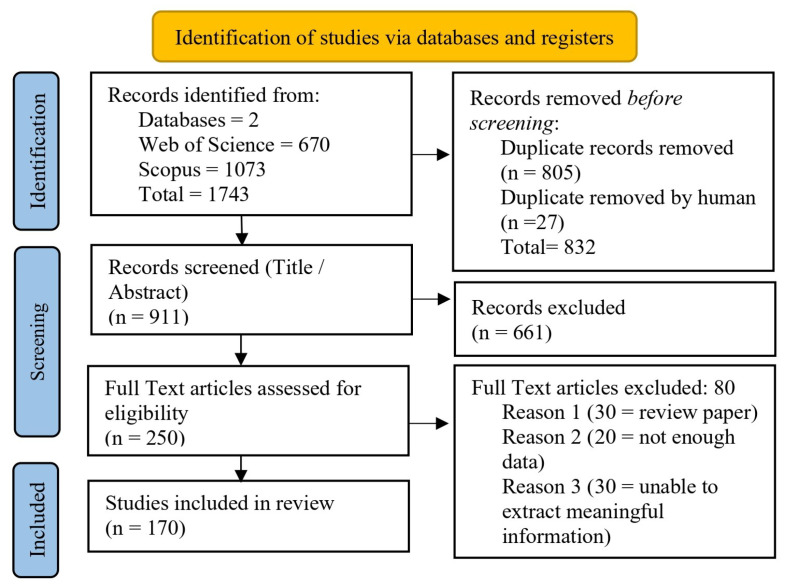
Flowchart of the methodology adopted for selecting papers included in this work, following PRISMA guidelines.

This study adopted a formal methodology for conducting systematic reviews following the PRISMA guidelines [26]. The methodology consists of several steps, which are detailed in Figure 2 and explained below.

A systematic search for relevant research articles for this review was conducted in two reliable electronic databases: Web of Science and Scopus. The search process included keywords with “OR” and “AND” operators, incorporating terms such as the following, with the intention of comprehensively identifying all possible and relevant research articles: (“UAV swarm” OR “drone swarm” OR “multi-UAV”) AND (“trajectory design” OR “path planning” OR “trajectory optimisation”) AND (“algorithm” OR “control” OR “strategy”).

A total of 1743 research articles were retrieved during this phase of the search. The authors then independently screened and selected these articles. Using Zotero 7 software, 832 articles were excluded as duplicate records, while 661 articles were excluded because they provided only a general overview and did not meet the study’s objectives. Therefore, only those articles that met the inclusion criteria were considered for review.

### 2.1. Screening of Articles

One author initially screened the research articles identified through the keyword search based on their titles and abstracts. A total of 911 studies were critically reviewed during this phase. All articles relevant to the topic of this study were included, while irrelevant studies were excluded.

If there was no consensus between the two authors regarding the selection or exclusion of a particular article, the entire article was carefully reviewed. If disagreement persisted, the final decision was made by a third, impartial reviewer to ensure transparency and objectivity.

### 2.2. Eligibility Criteria for Selection of Articles

This review included research articles that met the following criteria:

- The article used keywords such as “UAV swarm”, “drone swarm”, or “multi-UAV”.
- The article included research related to “trajectory design”, “path planning”, or “trajectory optimisation”.
- The article proposed a practical method or technique related to “algorithm”, “control”, or “strategy”.
- The research focused on issues such as collision avoidance, path optimisation, overlapping, and interference.
- The study should cover topics that are relevant to the practical application of UAV swarms.

This criterion is established to include only articles that focus on solving the problems of effective, safe, and practical UAV swarm trajectory design and control in real-world contexts.

### 2.3. Data Extraction Process

The extraction of information from the selected research articles is carried out in a systematic and standardised manner. For this purpose, a pre-prepared data extraction form is used, in which the following points are compiled from each study:

- Name of the author(s);
- Year of publication;
- Objective of the study;
- Method or algorithm used;
- Platform or simulator used;
- Key findings and recommendations of the study;
- Research limitations.

Data extraction is performed independently by two authors to minimise bias and ensure the accuracy of the results. In the event of any disagreement, the final decision is made after consulting with a third author. All the extracted information is compiled into a systematic table, which facilitates comparative analysis later.

### 2.4. Results and Analysis

A total of 250 research articles are ultimately included in this systematic review, as per our selection criteria of these 20 are review articles that helped us identify other research studies related to the topic [27,28,29,30,31,32,33,34,35,36,37,38,39,40,41]. Additionally, 75 articles are excluded because they do not meet the exclusion criteria.

The selected articles are divided into three main categories based on their research orientation: TA, BIAs, and modern AI-based approaches

The performance of the algorithms presented in each category is evaluated based on several standard metrics, including the following: overlapping and interference of paths, obstacle avoidance, and optimisation quality.

The study utilises various tables to present the performance of each algorithm or hybrid approach visually. In these tables, the performance of each approach is presented, allowing for easy comparison of different techniques.

## 3. Centralised Swarm vs. Decentralised Swarm

A swarm is a concept derived from nature, such as a flock of birds, a school of fish, or a colony of ants. It involves several autonomous units (agents) working both in a coordinated and uncoordinated manner, without any central control, and using only local information [42].

In the field of UAVs, a swarm refers to multiple drones or UAVs working together, communicating with each other, and operating under a collective goal, such as surveillance, search and rescue, or enemy identification [43,44]. There are two basic methods of controlling a swarm.

### 3.1. Centralised Swarm

**Figure 3:**
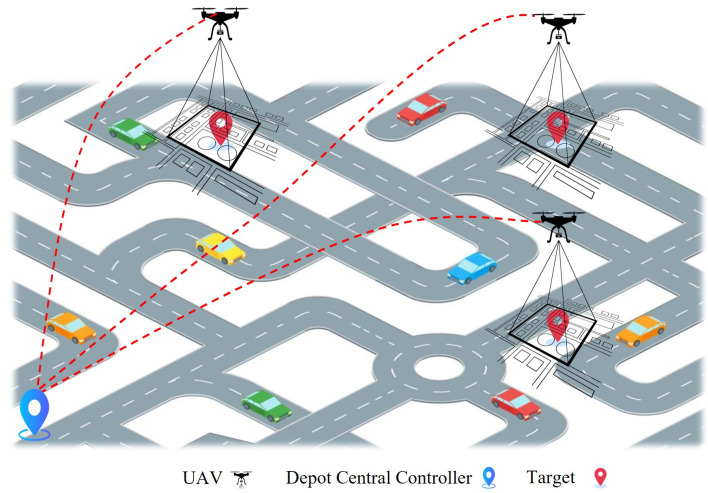
Illustration of a centralised controller.

A swarm of centrally controlled UAVs is a system in which all drones or UAV subunits are controlled by a single central system, such as a Ground Control Station or a cloud server, as depicted in Figure 3. This central station holds full responsibility for observation, control, and decision making. Each UAV receives specific instructions, tasks are distributed from this central unit, and each drone follows its defined flight plan or direction. Equation (1) presents the states of the centralised swarm.

(1) $$x_{i}\left(t+1\right)=x_{i}\left(t\right)+u_{c}\left(t\right),$$

where:

- $x_{i}\left(t\right)$ is the position of UAV, *i*, at time, *t*.
- $u_{c}\left(t\right)$ is the control signal sent to all UAVs by the centralised controller.

Examples of controlled centralisation have also emerged in both research and practical applications. The authors of [45,46] presented a comparative analysis of the performance of centralised control in a study, in which a cloud-based control system guided 12 drones cooperatively. The results showed that centralised control excels in decision making; however, scalability remains a significant challenge. Similarly, the authors of [47] introduced a centralised control-based hybrid AI system for ground surveillance. This system utilises Proximal Policy Optimisation (PPO)-based reinforcement learning models, where the centralised controller assigns specific search and tracking tasks to different UAVs. The results demonstrate that this centralised structure is effective for both search and continuous tracking.

The authors of [48] highlighted that the centralised task assignment mode is the most widely used, in which the Ground Control Station distributes tasks, and each UAV completes its flight. Although this improves the quality of decision making and ensures that the system follows a coherent strategy, as the number of UAVs increases, challenges such as network communication, real-time response capability, and computational scalability arise. Ultimately, the researchers who published [45,47] agree that centralised control has its advantages, but its challenges cannot be ignored.

### 3.2. Decentralised Swarm

**Figure 4:**
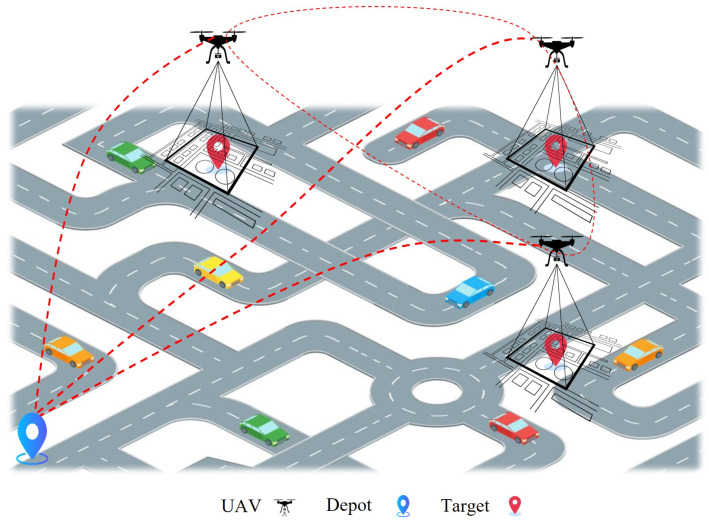
Illustration of a decentralised controller.

A decentralised UAV swarm is a model in which each UAV makes decisions based on its local information and signals received from neighbouring UAVs, as shown in Figure 4. In this approach, there is no “single point of failure”, meaning that, if a single UAV fails, the rest of the system continues to function. Various studies have highlighted the robustness and resilience of decentralised models. For example, the authors of [48,49] present UAV coordination models based on decentralised algorithms, which demonstrate the advantages of efficient, low-latency control using local information. This proves that decentralised structures are more suitable for UAV swarms where the communication network is limited or uncertain [50]. Equation (2) presents the coordination of the decentralised swarm:

(2) $$x_{i}\left(t+1\right)=x_{i}\left(t\right)+\sum_{j\in N(i)}a_{ij}\left(x_{j}\left(t\right)-x_{i}\left(t\right)\right),$$

where:

- $x_{i}\left(t\right)$ denotes the position of UAV, *i*, at time, *t*.
- N(i) is the set of UAVs neighbouring UAVs of UAV *i*.
- $a_{ij}$ is the magnitude of the influence that UAV *j* has on UAV*i*.

## 4. Trajectory Design vs. Path Planning

Although these two terms seem similar, there are several fundamental differences between them and their different uses have been repeatedly highlighted in research. For example, refs. [51,52,53] define path planning as a method that focuses primarily on finding the shortest path from a starting point to a target, while refs. [54,55,56,57] define trajectory design as the planning of a complete and safe flight path with time, velocity, and acceleration.

### 4.1. Path Planning

**Figure 5:**
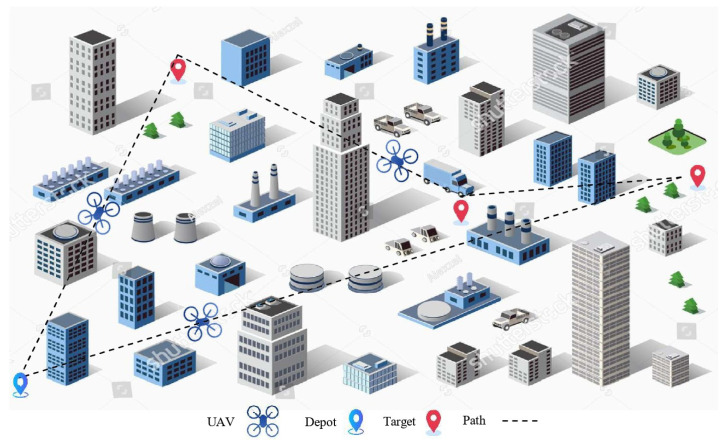
Illustration of path planning.

The goal of path planning is to find a path from a starting point to a destination with the shortest distance, as shown in Figure 5. This method is primarily used in static environments. It focuses on finding the shortest path based on local or global maps. Simple yet effective algorithms, such as those presented by Dijkstra [58] or A* [8], are used to obtain a path with the shortest distance.

The cost function, which is used to minimise the total length of the path, is given below:

(3) $$\min\sum_{i=1}^{n-1}∥p_{i+1}-p_{i}∥,$$

where:

- $p_{i}$ represents the waypoints of the path;
- $|p_{i+1}-p_{i}|$ is the distance between two consecutive points;
- *n* is the total number of points the path passes through.

### 4.2. Trajectory Designing

**Figure 6:**
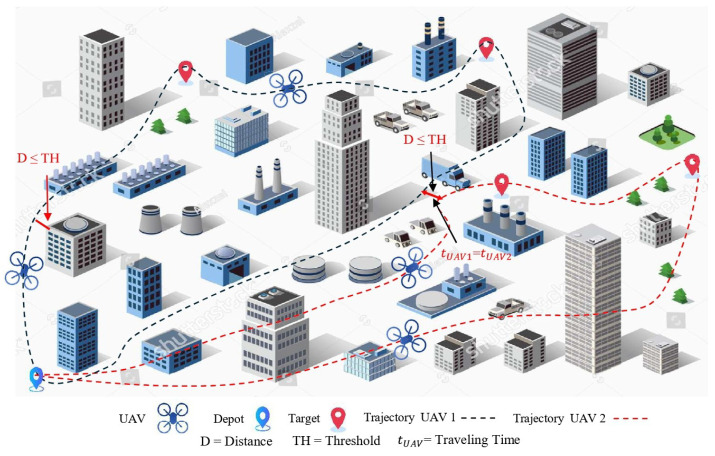
Illustration of trajectory planning/designing.

Trajectory design involves planning a fully dynamic flight path, including speed, time, angle, and acceleration as shown in Figure 6. This method is commonly used in autonomous drones and robots, where the flight must be not only accurate but also smooth and energy-efficient. For this purpose, a cost function is commonly used to minimise the flight speed and its change (acceleration) [27]. The function given below is based on this principle:

(4) $$J=\int_{0}^{T}\left(∥\dot{p}{\left(t\right)∥}^{2}+{∥\ddot{p}\left(t\right)∥}^{2}\right)dt,$$

where:

- $\dot{p}\left(t\right)$ is the velocity.
- $\ddot{p}\left(t\right)$ is the acceleration.
- *T* is the mission duration.

Table 1 provides a comparative overview of the main differences, application areas, and key technical aspects between path planning and trajectory designing. This comparison reveals that path planning is typically employed to find a safe path in a static environment. In contrast, trajectory design provides a smoother and more time-efficient path in a dynamic and uncertain environment, making it more flexible and better suited to modern UAV missions.

## 5. UAV Trajectory Design Issues and Use of MTSP

### 5.1. Nature of Problems and Solution Sequence

Trajectory design by UAV is a complex problem, especially when the target has to be reached at multiple locations, and the mission duration or energy is limited [59]. The following logical sequence is adopted to solve this problem:

1. Mission Definition: Target points, time limit, and objectives are specified.
2. Modelling: Targets are modelled as nodes, paths as edges, and distance/time as weights [60,61].
3. Problem Classification:
  - If there is one UAV, → TSP [62,63].
  - If there are multiple UAVs, → MTSP [64,65].
4. Trajectory Optimisation: A solution is derived using an appropriate heuristic or AI algorithm, which includes collision avoidance, energy limits, and other practical requirements [64,65].
5. Simulation or Practical Testing: the performance of the obtained solution is tested.

### 5.2. TSP and Its Application to UAVs

If there is only one UAV and it has to visit *n* destinations, the problem becomes the Travelling Salesman Problem (TSP) [62,63].

The objective of TSP is to visit all the destinations in the shortest distance or time and finally return to the starting point. The UAV path shown in Figure 5 is a practical example of solving the same TSP problem, where the UAV visits all the targets (waypoints) in a specific order to minimise the total distance.

(5) $$\min\sum_{i=1}^{n}\sum_{j=1}^{n}c_{ij}\cdot x_{ij},$$

where:

- $c_{ij}$ is the cost of travelling, i.e., time and distance from location *i* to *j*.
- $x_{ij}=1$ if the path is chosen.

### 5.3. When It Comes to Congestion: The Need for MTSP

TSP becomes inadequate in the presence of more than one UAV. Therefore, we use the MTSP, which assigns paths to multiple UAVs such that they collectively visit all the destinations in the shortest distance or time, and each UAV eventually returns to its starting point (Dpot) [64,65]. The UAV trajectory shown in Figure 6 is a practical example of solving the same MTSP problem, where each UAV visits a certain number of targets to minimise the total cost.

#### 5.3.1. Definition and Mathematical Model of MTSP

MTSP is an extended model, in which the following are true:

- *m* UAVs (salesmen);
- *n* targets (tasks or cities);
- Each target is assigned to only one UAV;
- All UAVs start and return from a depot.

Objective of MTSP:

(6) $$\min\sum_{k=1}^{m}\sum_{i=1}^{n}\sum_{j=1}^{n}c_{ij}\cdot x_{ijk},$$

where:

- $x_{ijk}=1$ if UAV *k* goes from location *i* to *j*.
- $c_{ij}$ is the distance or time value.
- *m* is the total number of UAVs.

#### 5.3.2. Application of MTSP to UAV Swarms

The use of MTSP in UAV swarms provides the following benefits:

- Parallelism: All UAVs perform separate missions simultaneously.
- Load Balancing: Fair distribution of targets is possible.
- Time Efficiency: The total mission time is reduced.
- Collision Avoidance: Obstacles are detected and avoided to ensure safe navigation.

While TSP is a suitable solution for UAVs, MTSP provides a very efficient, appropriate and workable framework for UAV swarms [60,61]. It not only improves speed but also enables missions to be completed in less time and with greater efficiency.

## 6. Different Trajectory Design Methods

Trajectory design is a complex problem, especially when it comes to UAVs or multi-agent systems. There are different strategies to solve this problem, which can be divided into three basic types.

### 6.1. Traditional Algorithms Used in UAV Swarms (In the Context of MTSP)

These TAs are usually used in static or known environments.

*Famous Algorithms:*
  - Dijkstra Algorithm [7];
  - A* Algorithm [66];
  - Rapid Random Tree Search (RRT) [67];
  - Dynamic Window Approach (DWA) [68];
  - Dubins Path [9].

UAV swarm-based trajectory design leverages TAs to identify optimal and safe paths to targets. In MTSP scenarios, these algorithms efficiently generate individual UAV trajectories, as illustrated in Algorithm 1, which depicts TAs’ operations.
**Algorithm 1** UAV swarm trajectory design using MTSP.1:**Input:**$V=\{v_{0},v_{1},\ldots ,v_{n}\}$ (locations, with $v_{0}$ as the base station);$U=\{u_{1},u_{2},\ldots ,u_{m}\}$ (set of UAVs);$C(v_{i},v_{j})$ (cost between locations).2:**Output:** Paths $P_{k}$ for each UAV, such that:$$\min\sum_{k=1}^{m}\sum_{\left(i,j\right)\in P_{k}}C\left(v_{i},v_{j}\right),\;P_{i}\cap P_{j}=\left\{v_{0}\right\},\forall i\neq j$$3:**Initial Step:** Set starting location $v_{0}$ for each UAV, and mark all $v_{i}\in V$ as unvisited (except $v_{0}$).4:**while** Unvisited nodes exist in *V* **do**5:    **for** each UAV $u_{k}$ **do**6:        Select the nearest unvisited node:$$v_{\mathrm{next}}=\arg\min_{v_{j}\in V_{\mathrm{unvisited}}}C\left(\mathrm{current}\left(u_{k}\right),v_{j}\right)$$7:        Add $v_{\mathrm{next}}$ to path $P_{k}$ and mark it as visited8:    **end for**9:**end while**10:**Return:** UAVs return to base $v_{0}$, with final paths $P_{k}$.

#### 6.1.1. Dijkstra Algorithm and Its Role in UAV Swarms

Dijkstra’s algorithm is a classic graph search technique that finds the least-cost or shortest path from one point to all other points. It is beneficial in UAV trajectory design once the MTSP has been solved, as it provides an efficient and shortest path for each UAV to reach its assigned targets [7]. Thus, this algorithm helps to reduce both the time and total cost of mission completion. This concept can be expressed mathematically as:

(7) $$\min\sum_{\left(i,j\right)\in P_{k}}c_{ij},$$

where:

- $P_{k}$: path of UAV *k*;
- $c_{ij}$: cost of travelling from node *i* to node *j*.

Research on UAV path planning has proposed basic algorithms that typically determine the optimal path from a cost map in a static 2D or 3D grid environment, yielding effective results in simple scenarios. However, these methods are generally limited to single-UAV operations and cannot coordinate large-scale UAV swarms [69]. In the same vein, another study designed a pathfinding model for a group of 3–10 UAVs, taking into account battery limits, charging stations and coverage constraints, which provides more effective coverage and better mission completion. However, path overlap remains a key challenge [70]. In another study, the initial paths obtained from classical Dijkstra are improved by PSO to enhance collision avoidance and path selection, resulting in better performance in complex scenarios with reduced path overlap and outperforming classical Dijkstra [58]. Additionally, dynamic-planning-based methods, which utilise local replanning with the Bresenham algorithm, have been proposed to avoid unknown obstacles in both static and dynamic environments. They are mainly effective for single UAVs and are capable of handling sudden changes and new obstacles [7]. Although Dijkstra-based method provides reliable routing for UAVs, classical Dijkstra has problems such as synchronization and lack of coordination of large-scale UAV swarms, which make it inadequate for large systems; however, its modern variants such as multi-UAV Dijkstra and Dijkstra + PSO [58] overcome these weaknesses and provide more reliable solutions within UAV swarms with better coverage, effective collision avoidance, and less interference.

#### 6.1.2. A* Algorithm

The A* algorithm is a heuristic-guided version of Dijkstra, which uses the heuristic function h(n) to speed up the search process [71]. It considers the least-cost path, as well as the estimated remaining distance, in the graph-based search, making it more computationally efficient than the classical Dijkstra algorithm. The cost function in A* algorithm can be expressed as:

(8) f(n)=g(n)+h(n),

where:

- g(n): actual cost of reaching node *n*;
- h(n): heuristic estimate of remaining distance.

The TA grid-based A* algorithm is utilised for UAV scheduling and routing, providing efficient coordination of 3–10 UAVs while minimising mission overlap through temporal offset batching. To improve upon this, Jump Point Search (JPS)-Enhanced A is introduced, which finds faster paths by skipping unnecessary nodes and gives better results in environments with static obstacles. However, some path overlap is reported during Moving Window Search [72]. As a further development, the 3D A algorithm provided efficient navigation in complex three-dimensional environments using octree-based space partitioning and reduced collisions through per-UAV deflection layers. Still, its performance remained relatively limited in unpredictable dynamic scenarios [73]. In the same sequence, Classification A implemented local A on each UAV by dividing the workspace into sectors, which reduced the computing time and achieved better results [74]. Overall, A* and its variants provide fast, reliable, and effective solutions for UAV trajectory design; however, challenges such as scalability and limited replanning capacity in large-scale UAV swarms and highly dynamic environments remain, which require more hybrid and adaptive approaches to overcome.

#### 6.1.3. Rapidly-Exploring Random Trees (RRT)

RRT is a sampling-based path planning algorithm that rapidly grows new branches through random sampling in a given configuration space, to explore as much accessible space as possible [67,75]. The following function is used to select the nearest node and extend it in a randomly chosen direction:

(9) $$x_{\mathrm{new}}=x_{\mathrm{near}}+\epsilon \cdot \frac{x_{\mathrm{rand}}-x_{\mathrm{near}}}{∥x_{\mathrm{rand}}-x_{\mathrm{near}}∥},$$

where:

- $x_{\mathrm{near}}$: current node in the tree that is closest to $x_{\mathrm{rand}}$;
- $x_{\mathrm{rand}}$: randomly chosen point in the direction in which the tree is expanded;
- ϵ: step size that determines the extent of the expansion;
- $∥x_{\mathrm{rand}}-x_{\mathrm{near}}∥$: Euclidean distance between the two points, which normalises the direction.

The initial research utilises Multi-platform Space–Time RRT, which enables UAVs to operate in static and cluttered 3D environments with space and time constraints. This model provides smooth and flyable paths, where path overlap is significantly reduced by strictly enforcing the time and separation of each UAV. Another study [76] adopted multi-RRT with kinodynamic constraints and Bézier curves, which not only provided smoother and shorter paths for 3–10 UAVs but also improved upon methods such as classical RRT and Theta-RRT [77], while ensuring collision avoidance. Meanwhile, RRT is utilised for single-UAV scenarios in photogrammetry and aerial survey, where real-time obstacle avoidance is possible with the aid of stereo cameras, and safe navigation at speeds of 6 m/s is demonstrated in practical missions [78]. Furthermore, a hybrid method is introduced that combines iterative RRT with the Salp Swarm Algorithm (SSA), in which SSA intelligently guides the expansion of nodes. This approach reduces path length, decreases the number of iterations and nodes used, improves computational efficiency, and further minimises overlap between UAV paths [79]. Overall, RRT-based algorithms are highly effective in UAV trajectory planning, particularly in complex, dynamic, or partially known environments. Their main strength is fast search; however, the randomness and non-smooth nature of classical RRT often create limitations, which is why modern research is integrating these techniques with Bézier smoothing or SSA-guided approaches to enable smoother, collision-free, and computationally efficient trajectories for UAV swarms.

#### 6.1.4. Dynamic Window Approach (DWA)

DWA is a real-time spatial planning algorithm that selects a safe and feasible path within the UAV’s current velocity *v* and angular velocity (ω). This method is effective because it enables the UAV to avoid collisions even in rapidly changing conditions and complex or partially unknown environments. It analyses possible movements based on velocity and angle, assesses the safety and feasibility of each path, and instantly selects the path that provides the least risk and the most efficiency [80,81]. In DWA, the objective function is used to select the optimal path, considering various factors such as target alignment, obstacle distance, and speed. Its mathematical expression is as follows:

(10) G(v,ω)=α·heading+β·distance+γ·speed,

where:

- *v*: velocity, ω: angular velocity;
- heading: target alignment;
- distance: distance from the obstacle;
- speed: current speed of the UAV;
- α,β,γ: weights that describe the relative influence of heading, clearance, and speed in decision making.

This function (Equation (10)) combines these parameters to produce a score for each possible move, based on which the most suitable move is selected.

DWA has been adopted in various scenarios in UAV swarms to enable quick response and collision avoidance. Several studies have shown that DWA-based approaches not only make the routes safer during missions but also significantly improve the overall efficiency of UAVs. For example, the authors of [82] combined DWA with ORCA (Optimal Reciprocal Collision Avoidance), resulting in a 17% reduction in mission time and a 27.9% reduction in path length. A study [83] utilised DWA with gradient-field costs to enable UAVs to navigate more effectively around non-convex obstacles, although gradient sensitivity occasionally led to local minima. Similarly, the authors of [84] combined DWA with global planners such as Jump Point Search (JPS), where the combination of local collision avoidance and global route guidance provided smoother paths. Overall, DWA is a reliable method for real-time local motion planning, enabling UAVs to make swift decisions in dynamic and partially known environments. It provides collision-free trajectories in a short time and improves mission duration. However, for large-scale coordination and nonlinear interactions in complex UAV swarms, DWA typically requires integration with global planners or AI-based intelligence to provide more scalable and adaptive solutions.

#### 6.1.5. Dubins Path

Dubin’s path is a classical geometric trajectory planning model designed for vehicles with limited turning radius, and is particularly suitable for fixed-wing UAVs where zero-radius turns are not possible [9,85]. The model searches for a minimum path that consists of only three basic movements: straight ahead (*S*), left turn (*L*), or right turn (*R*). The combinations of these movements create different possible paths, which can be expressed mathematically as:

(11) Path={LSL,LSR,RSL,RSR,RLR,LRL},

This set represents all the basic possible paths that the model evaluates for minimum distance or cost. In this way, the model compares the performance of each combination and selects the most efficient route, which saves both time and energy in UAV navigation and path planning.

The Dubins path model has been adopted in several studies in UAV trajectory planning. The authors of [86] developed a Dubins-based motion planning framework for fixed-wing UAVs, which is found to be effective for constrained turns and short-path planning. Another study in [87] designed minimum-turn paths for UAVs, which improve trajectory smoothness and mission efficiency in different environments. Furthermore, the authors of [88] integrated Dubins paths into cooperative UAV swarms, providing collision-free trajectories in a multi-agent path-planning scenario despite the turning constraints.

Dubin’s path model is a crucial technique for fixed-wing UAV swarms because it incorporates physical constraints, like turning radius, directly into the trajectory planning process. However, this model has limitations; it can only handle straight, constant-radius turns, making it less suitable for dynamic replanning. Therefore, it is often integrated with advanced methods or hybrid approaches in more complex scenarios.

Table 2 presents a comparative overview of different TAs in MTSP, showing that each algorithm plays a unique role in specific environments and scenarios. Observations indicate that a combination of different algorithms yields more effective, flexible, and situationally superior results in UAV swarm missions.

### 6.2. Bio-Inspired Methods Used in UAV Swarm

BIAs are inspired by simple yet effective behaviours found in nature. These heuristic-based methods are highly effective in solving NP-hard problems, such as the MTSP, particularly when designing trajectories for UAV swarms. As demonstrated in Algorithm 2, this illustrates the operational framework of TAs. Some famous algorithms are as follows:

- Pigeon-Inspired Optimisation (PIO) [89].
- Salp Swarm Algorithm (SSA) [90].
- Artificial Bee Colony (ABC) [16].
- Ant Colony Optimisation (ACO) [14].
- Particle Swarm Optimisation (PSO) [13].
- Genetic Algorithm (GA) [15].

**Algorithm 2** General flow of BIAs for UAV swarm and MTSP.1:**Input:**$V=\{v_{0},v_{1},\ldots ,v_{n}\}$: Hotspots (where $v_{0}$ is the base station)$U=\{u_{1},u_{2},\ldots ,u_{m}\}$: Set of UAVs$C(v_{i},v_{j})$: Cost (distance, time, or energy) between locationsAlgorithm-specific parameters (e.g., pheromone τ for ACO, velocity *v* for PSO, etc.)2:**Output:** Optimal paths $\left\{P_{1},P_{2},\ldots ,P_{m}\right\}$ that minimize the total cost:$$\min\sum_{k=1}^{m}\sum_{\left(i,j\right)\in P_{k}}C\left(v_{i},v_{j}\right),$$
with each city visited by only one UAV (except the base station).3:**Initial step:**4:Create an initial population/colony/cluster for each BIA:$$Pop=\{Sol_{1},Sol_{2},\ldots ,Sol_{p}\},$$Where each solution is a set of possible paths for the UAVs.5:Set initial algorithm parameters (pheromone level, inertia weight, learning coefficients, etc.).6:**while** termination criterion is not met (e.g., max iterations or convergence) **do**7:    **for** each solution $Sol_{i}\in Pop$ **do**8:        Calculate fitness:$$f\left(Sol_{i}\right)=\sum_{k=1}^{m}\sum_{\left(i,j\right)\in P_{k}}C\left(v_{i},v_{j}\right)$$9:        Update pheromone (for ACO):$$\tau_{ij}\leftarrow \left(1-\rho \right)\tau_{ij}+\Delta \tau_{ij}$$10:        Update velocity and position (for PSO):$$v_{i}\left(t+1\right)=\omega v_{i}\left(t\right)+c_{1}r_{1}\left(pbest-x_{i}\right)+c_{2}r_{2}\left(gbest-x_{i}\right)$$11:        Apply selection, crossover, mutation (for GA).12:    **end for**13:    Update best solution (global best or optimal).14:**end while**15:**Output:** Extract best solution $\left\{P_{1},P_{2},\ldots ,P_{m}\right\}$, providing optimal or near-optimal MTSP paths for UAVs.

#### 6.2.1. Pigeon-Inspired Optimisation (PIO)

PIO is a BIA based on the navigation abilities, memory, and tendency of pigeons to use the Earth’s magnetic field. PIO can be used to navigate UAVs in the right direction toward the global target, providing speed and accuracy in path planning. The algorithm was first introduced by the authors of [89], who described it in two main steps: the map and compass operator, inspired by pigeons’ direction recognition and magnetic sensing, and the landmark operator, which reflects pigeons’ memory and ability to fly to a target.

In recent research, PIO has been applied to various engineering and optimisation problems. The authors of [91] applied PIO to UAV path planning and showed that it can derive paths to the target in less time than TAs. Similarly, Sharma and Panda [92] used PIO in multiobjective trajectory design, where PIO struck a balance between collision avoidance and energy efficiency. Furthermore, the authors of [93] adapted PIO for UAV swarms to provide effective navigation toward the global target even in dynamic and uncertain environments. In the UAV swarm MTSP scenario, the compass-based formula in PIO is used to guide each UAV to the global best position ($x_{g}$). This enables coordinated movement of UAVs and efficient multi-target allocation. This method minimises the total travel distance while maintaining swarm coordination and ensuring the avoidance of unnecessary or redundant paths. This compass-based update formula is mathematically expressed as:

(12) $$x_{i}^{t+1}=x_{i}^{t}\cdot e^{-Rt}+x_{g},$$

where:

- $x_{g}$: global best position;
- *R*: learning rate that reduces the intensity of the movement over time.

This Equation (12) ensures that over time, each UAV gradually moves from its current position to the global optimal position, allowing the entire swarm to complete the MTSP mission in a coordinated and efficient manner.

#### 6.2.2. Salp Swarm Algorithm (SSA)

The Salp Swarm Algorithm (SSA) is a bio-inspired optimisation method inspired by the movement of a swarm of salps in the ocean, where a leader salp moves towards a target and the rest of the salps follow it. SSA is first introduced by the [90], and consists of two stages: the movement of the leader salp that controls the exploration, and the movement of the follower salp that fine-tunes the exploitation.

SSA has demonstrated its effectiveness in various engineering applications over the past few years. For example, [94] utilised SSA for UAV path planning and showed that it can identify the most efficient paths even in complex and dynamic environments. Similarly, the authors of [95] implemented SSA in multiobjective optimisation, where energy consumption and path length are optimised simultaneously. Furthermore, the authors of [96] extended SSA to complex problems, such as UAV swarm coordination and MTSP, and demonstrated its flexibility.

Leader swarm update equation:

(13) $$x_{1}^{j}=\left\{\begin{array}{cc} F_{j}+c_{1}\left(\left(ub_{j}-lb_{j}\right)c_{2}+lb_{j}\right), & c_{3}\geq 0.5\\ F_{j}-c_{1}\left(\left(ub_{j}-lb_{j}\right)c_{2}+lb_{j}\right), & c_{3}<0.5 \end{array}\right.$$

Leader swarm update equation components:

- $x_{1}^{j}$: new position of the leader swarm in dimension *j*;
- $F_{j}$: position of the target (food source) in dimension;
- $ub_{j}$: upper bound in the given dimension;
- $lb_{j}$: lower bound in the given dimension;
- $c_{1}$: exploration coefficient, which decreases with time;
- $c_{2}$, $c_{3}$: random numbers between 0 and 1.

- If $c_{3}\geq 0.5$, the swarm moves towards the target.
- If $c_{3}<0.5$, the swarm moves away from the target, which maintains diversity.

In SSA, the movement of the leader swarm controls the overall direction and behaviour of the entire swarm. In the context of a UAV swarm, the leader swarm can be a UAV that determines the general movement of the swarm towards the target, while the rest of the UAVs follow it. This mechanism is considered ideal for maintaining a balance between exploration and exploitation in complex path planning problems, such as MTSP.

#### 6.2.3. Artificial Bee Colony (ABC)

ABC is a popular bio-inspired optimisation algorithm inspired by the natural foraging behaviour of honeybees. The authors of [97] introduced ABC, which consists of three types of bees employed: onlooker, scout, and worker bees. Each bee plays a role in the process of finding new food (solutions), exchanging information, and making better choices. The ABC algorithm has been successfully applied to various complex problems in engineering and robotics. For example, ref. [16] uses it for numerical optimisation, while [98] shows in UAV path planning that crowd-based cooperation accelerates the search for better paths. In the same vein, ref. [99] applied the ABC approach to multiobjective optimisation in UAV swarms, where the optimal speed and path are determined while considering constraints such as energy, time, and distance.

These studies present the current state of the problem and possible search paths, illustrating that each UAV requires both local and global information to determine the optimal direction. This concept is mathematically represented in the following equation, which is the basic formula for generating a new solution:

(14) $$v_{ij}=x_{ij}+\phi_{ij}\left(x_{ij}-x_{kj}\right),$$

where:

- $x_{ij}$: current solution (the current path or speed of the UAV);
- $x_{kj}$: neighbouring solutions (other UAVs or alternative paths);
- $\phi_{ij}$: a random value that diversifies the search.

This update mechanism, as explained in Equation (14), describes how each UAV combines its current state with neighbouring information to generate a new solution. By applying this equation, improved paths and speeds are achieved, providing fast and effective solutions to complex problems, such as the MTSP. This enables each UAV to determine the optimal path or trajectory in a cooperative manner. The collective intelligence of the UAVs yields faster and more efficient solutions to complex problems.

#### 6.2.4. Ant Colony Optimisation (ACO)

ACO is another important BIA inspired by the natural path-finding behaviour of ants, where ants leave pheromone trails and use them to find the best path. The authors of [100] founded ACO, and it remains a benchmark method for many optimisation problems today.

The authors of [101] utilised ACO for cooperative search and surveillance missions in UAVs, demonstrating that pheromone-based learning enables effective navigation for UAVs even in dynamic environments. Furthermore, the authors of [102] modified ACO to solve UAV-based MTSP and observed that it provides better scalability in parallel UAV coordination.

In MTSP, each UAV is considered as an “ant” searching for the best possible path to reach its target. The initial state of the problem, including all possible paths, as each UAV explores different paths. In this search process, each UAV learns from its own and other UAVs’ previous movements to choose the best path for the future. The following probability equation decides this selection:

(15) $$P_{ij}=\frac{{\left[\tau_{ij}\right]}^{\alpha }{\left[\eta_{ij}\right]}^{\beta }}{\sum_{k\in \mathrm{allowed}}{\left[\tau_{ik}\right]}^{\alpha }{\left[\eta_{ik}\right]}^{\beta }},$$

where:

- $\tau_{ij}$: pheromone level, which indicates the previous success of a path;
- $\eta_{ij}$: approximate information (1/distance), which gives the immediate availability of the route.

Equation (15) helps each UAV calculate which of the following cities or targets is most suitable to choose. The probability of selecting a route with a higher pheromone level and shorter distance increases, while the probability of choosing a path with a lower pheromone level and longer distance decreases.

ACO’s pheromone trails provide UAVs with a “collective memory”, which is updated after each iteration. This means that, when a UAV passes a good route, it leaves pheromones along that route, which other UAVs sense and incorporate into their decisions. This collaboration results in the emergence of optimal routes in the final graph, where each UAV reaches its assigned targets in the shortest distance, time, and energy.

In this sequence, the initial state → conducts decision making through equations → in which the pheromone updates the → optimised paths, helps solve complex problems like the MTSP efficiently and consistently.

#### 6.2.5. Particle Swarm Optimisation (PSO)

PSO is a popular bio-inspired metaheuristic algorithm inspired by the collective behaviour of flocks of birds and schools of fish. The authors of [103,104] introduced PSO, in which each possible solution is considered a “particle” that explores the solution space by continuously updating its velocity and position.

In recent years, PSO has been widely adopted in UAV path planning and swarm coordination problems. The authors of [105] utilised PSO in the trajectory optimisation of UAVs and demonstrated that the algorithm quickly finds near-optimal paths, even in dynamic environments. Similarly, the [106] implemented PSO in UAV-based multi-target assignment (MTSP) and observed that this approach provides better load balancing while maintaining a low computational cost. Furthermore, the authors of [107] used an improved version of PSO in UAV swarm collision avoidance, and the results showed that PSO-based coordination is effective in both safety and efficiency.

In MTSP, each particle represents a possible path or velocity and learns from its personal best and the group’s global best. The following equation controls the velocity update:

(16) $$v_{i}^{t+1}=\omega v_{i}^{t}+c_{1}r_{1}\left(p_{i}-x_{i}^{t}\right)+c_{2}r_{2}\left(g-x_{i}^{t}\right),$$

where:

- $\omega v_{i}^{t}$: inertial component—maintains the current direction and velocity;
- $c_{1}r_{1}\left(p_{i}-x_{i}^{t}\right)$: cognitive component—movement towards the personal best position $p_{i}$;
- $c_{2}r_{2}\left(g-x_{i}^{t}\right)$: social component—movement towards the collective best solution *g*;
- $c_{1}$, $c_{2}$: learning coefficients;
- $r_{1}$, $r_{2}$: random factors that diversify the search.

The following equation then updates the position:

(17) $$x_{i}^{t+1}=x_{i}^{t}+v_{i}^{t+1},$$

where:

- $x_{i}^{t}$: current position;
- $v_{i}^{t+1}$: newly updated velocity.

Together, these two Equations (16) and (17) show a process in which each UAV continuously improves its position and velocity, first by taking advantage of its own experience and then by taking advantage of the collective experience of the group. Thus, the collective intelligence of PSO facilitates the identification of optimal paths in MTSP, utilising the minimum distance, time, and energy, and enables real-time swarm coordination.

#### 6.2.6. Genetic Algorithm (GA)

GA are a popular evolutionary optimisation technique based on the principles of natural evolution, such as selection, crossover, and mutation. Goldberg [15,18] introduced GA as a general framework for complex optimisation problems. Since then, GA has been widely used in various fields, including robotics and UAV path planning.

GA has repeatedly proven its usefulness in UAVs and swarm operations. The authors of [108,109] utilised GA for UAV mission planning and demonstrated how chromosome-based encoding reduces the total cost (in terms of time and distance) by optimising multiple paths. Furthermore, the researchers who published [110] utilised GA for UAV trajectory optimisation in the context of the MTSP, which demonstrated significant improvements in load balancing and mission completion time among UAVs. Similarly, the studies [111,112] implemented GA in UAV-based collision-free path planning, and the results showed that GA-based approaches remain efficient and scalable even in large search spaces.

In MTSP, the GA represents each possible UAV path as a chromosome, where genes represent the sequence of cities or targets that the UAV can visit. The goal of the GA is to find the solution among these paths that provides the least cost (distance or time). The following fitness function is used to measure this performance:

(18) $$Fitness\left(x\right)=\frac{1}{\mathrm{Cost}(x)},$$

where:

- Cost(x): total cost of the UAV path, measured in terms of distance or time.

Equation (18) ensures that the lower the cost of the path, the higher its fitness. As a result, the GA naturally prefers low-cost and high-fitness paths.

The GA iteratively generates new solutions:

1. Crossover: creates a new path by combining two existing paths.
2. Mutation: creates diversity by making minor changes to the path.
3. Selection: selects paths with better fitness for the next generation.

With each iteration, weaker solutions are eliminated and stronger solutions become more dominant, until all UAVs agree on an optimal or closest solution. The final part presents the results of this evolutionary process, where non-conflicting and low-cost paths for the UAVs emerge. Thus, GA’s evolutionary search enables the solution of complex problems, such as MTSP, quickly and efficiently, whether the problem involves trajectory planning, path allocation, or real-time swarm coordination.

### 6.3. Challenges in Bio-Inspired Algorithms

In the context of UAV swarms, several BIAs have been effectively adopted to solve complex combinatorial problems such as the MTSP. A specific natural phenomenon or organism inspires each algorithm, which then performs in UAV swarms with its unique mechanisms and advantages. However, each algorithm also has some limitations, which subsequent methods aim to address and improve. Table 3 summarises these algorithms, describing the basic motivation of each algorithm, its role in UAV swarms/MTSP, and the main challenges.

This evolutionary sequence illustrates that each new algorithm overcomes the weaknesses of its predecessors to some extent. For example, PIO relies on basic GPS-like navigation behaviour; however, it often fails to reach the global optimum. This shortcoming is partially addressed by SSA, which introduced a simple leader–follower strategy; however, it also proved to be limited in more complex and dynamic environments.

Then, ABC improved exploration by modelling the foraging activity of worker bees; however, it took longer in large search spaces due to slow convergence. ACO introduced collective learning through cooperative pheromone trails; however, it suffered from problems such as premature convergence and pheromone evaporation.

PSO provided an effective yet simple coordination mechanism by combining individual and collective best (personal best and global best). Still, it often became stuck in local minima due to the difficulty in maintaining diversity. Finally, GA emerged with an evolutionary mechanism that provides substantial diversity through crossover and mutation, offering a highly reliable and robust solution to complex combinatorial problems, such as MTSP [18,110].

However, a fundamental limitation of GA is that it is primarily suited for offline scenarios, where all the data is already available. In online situations such as real-time UAV coordination, the computational complexity and latency of GA can limit quick decision making. Therefore, while GA performs well in offline mission planning, either lightweight algorithms or hybrid approaches may be more effective for online decision making [105,111,113].

### 6.4. AI-Based and Innovative Methods

In recent years, artificial-intelligence-based methods have emerged as a crucial alternative for solving complex combinatorial problems, such as UAV swarm trajectory design and the MTSP. These innovative approaches have provided more adaptive, scalable, and data-driven solutions than TAs. AI-based algorithms enable UAVs to make autonomous decisions in changing environments and derive optimal routes in complex situations [114,115].

*Popular AI-based methods:*
  - Multi-Agent Reinforcement Learning (MARL) [116].
  - Deep Reinforcement Learning (DRL) [117].
  - Q-Learning/Deep Q-Network (DQN) [118].
  - Actor–Critic Methods [115].
  - Imitation Learning [119].
  - Active Inference [120].

These AI-based approaches have ushered in a new era for UAV-based MTSP and trajectory planning, where UAVs not only operate according to pre-programmed rules but also adapt and perform effectively in complex, real-world scenarios, with the ability to learn and make autonomous decisions. As shown in Algorithm 3, this outlines the operational framework of AI-based methods for UAV swarm.
**Algorithm 3** AI Techniques for UAV Swarm and MTSP1:**Input:**$V=\{v_{0},v_{1},\ldots ,v_{n}\}$: Hotspots (with $v_{0}$ as the base station)$U=\{u_{1},u_{2},\ldots ,u_{m}\}$: Set of UAVs$C(v_{i},v_{j})$: Cost (distance, time, or energy) between locationsAI-specific parameters (e.g., learning rate, neural network structure, etc.)2:**Output:** Optimal paths $\left\{P_{1},P_{2},\ldots ,P_{m}\right\}$ that minimize the total cost:$$\min\sum_{k=1}^{m}\sum_{\left(i,j\right)\in P_{k}}C\left(v_{i},v_{j}\right),$$
with each city visited by only one UAV (except the base station).3:**Initial Step:**4:Initialise neural network weights, or reinforcement learning environment.5:Set starting locations for each UAV $v_{0}$.6:**while** Not converged (e.g., max epochs, acceptable error) **do**7:    **for** each UAV $u_{k}$ **do**8:        Input current state (current location, previous path, etc.) into the AI model.9:        Output next location for UAV:$$v_{\mathrm{next}}=\mathrm{AI}\_\mathrm{model}\left(state\right)$$10:        Add $v_{\mathrm{next}}$ to UAV path $P_{k}$.11:        Update model parameters based on the UAV’s decision (Reinforcement Learning: update Q-value or loss function).12:    **end for**13:**end while**14:**Return:** Extract best solution $\left\{P_{1},P_{2},\ldots ,P_{m}\right\}$, providing optimal or near-optimal MTSP paths for UAVs.

#### 6.4.1. Multi-Agent Reinforcement Learning (MARL)

MARL is an extension of traditional reinforcement learning in which multiple agents learn and act together in the same environment [116,121]. In MARL, each agent not only receives rewards and observations from the environment, but is also influenced by the presence and decisions of other agents. This feature is particularly suitable for UAV swarms because each UAV acts as an agent that determines its trajectory and decisions by taking into account the behaviour of other UAVs.

In recent years, MARL has been widely used for UAV swarm trajectory planning, MTSP, and cooperative decision making. For example, the study [122] proposed a MARL-based framework for UAV swarms, which enables UAVs to jointly find optimal routes and share tasks (i.e., task allocation). Similarly, the authors of [123] used a MARL model based on centralised training and decentralised execution (CTDE) for UAV collision avoidance, which provides better coordination in real time decisions. Furthermore, the authors of [124] demonstrated that MARL enables UAVs to be cooperative and adaptive in dynamic MTSP scenarios, particularly in environments where targets and routes change over time.

**Figure 7:**
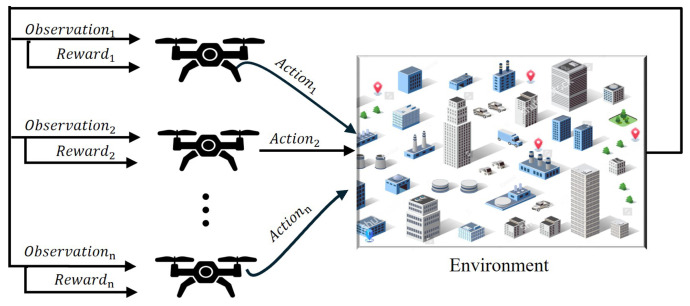
Example of a MARL framework for UAV trajectory planning.

Figure 7 illustrates the concept of multi-agent reinforcement learning (MARL), where each UAV makes autonomous decisions based on its local observations and the rewards it receives. Each UAV learns not only from its own experience but also from the behaviour of other UAVs, allowing for better collective decision making. This process can be described mathematically by the following objective function:

(19) $$\pi_{i}^{*}=\arg\max_{\pi_{i}}E\left[\sum_{t=0}^{T}\gamma^{t}r_{i,t}\;|\;\pi_{1},\pi_{2},...,\pi_{n}\right],$$

where:

- $\pi_{i}$: policy of agent *i*, which chooses an action based on current observations;
- $r_{i,t}$: reward received by agent *i* at time *t*;
- γ: discount factor, which maintains the importance of long-term rewards;
- $\pi_{1},\pi_{2},\ldots ,\pi_{n}$: policies of all other agents, which influence the environment and decisions.

Equation (19) specifies that each UAV optimises its policy in such a way that the long-term total reward is maximised, while also taking into account the behaviour of other UAVs.

The MARL’s frequently updated decisions enable UAVs to learn from each other, taking paths that avoid collisions, reduce time and distance, and successfully solve complex problems, such as MTSP, in dynamic and uncertain environments.

Thus, MARL provides an effective solution for UAV swarms, enabling them to adapt in real time and collectively adopt the best strategy [42,124].

#### 6.4.2. Deep Reinforcement Learning (DRL)

DRL is a modern learning method where an agent observes the environment, performs actions, and improves its policy based on rewards [20,125]. DRL combines the principles of classical reinforcement learning with deep neural networks, allowing it to learn efficiently even on high-dimensional inputs such as images, sensor data, and complex state spaces.

DRL has been widely used in complex combinatorial optimisation problems such as UAV trajectory design and MTSP. For example, the article [126] proposes a policy framework based on DRL for UAV swarms, allowing UAVs to perform dynamic task allocation and real-time trajectory adjustments. Similarly, the study [114] demonstrated that DRL enables UAVs to make adaptive routing decisions in response to changing situations during mission execution. Furthermore, the research presented in [22] achieved significant improvements in both load balancing and mission completion time by implementing DRL in an MTSP setting. This process can be described mathematically by the following objective function:

(20) $$\pi^{*}=\arg\max_{\pi }E\left[\sum_{t=0}^{T}\gamma^{t}r_{t}\right],$$

where:

- π: policy that describes the strategy for choosing the action;
- $r_{t}$: reward received at time *t*;
- γ: discount factor that balances long-term and short-term rewards.

Equation (20) explains that in DRL, the UAV optimises its policy π in such a way that the long-term total reward is maximised. After each observation, the UAV estimates which action in the current state will yield the most benefit in the future and updates its decisions accordingly.

The result of this iterative process is that the UAVs have learned from the environment and adopted better paths and target preferences for the MTSP. This has not only increased mission performance but also reduced execution time. Thus, DRL enables UAV swarms to operate effectively in dynamic and uncertain environments and automatically select the best paths [115,117].

#### 6.4.3. Q-Learning/Deep Q-Network (DQN)

**Figure 8:**
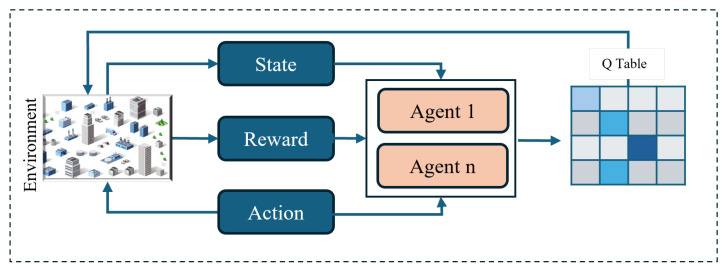
Illustrative example of Q-Learning/DQN approach for UAV-based MTSP.

Q-Learning is a classical value-based reinforcement learning technique that learns the expected reward for each state-action pair and ultimately produces an optimal policy [127]. Figure 8 demonstrates the fundamental framework of Q-Learning and DQN. After receiving state and reward from the environment, agents update the Q-Table to learn which action is best in which state, and this knowledge helps to improve subsequent decisions. The principle of this update is described in the following equation:

(21) $$Q\left(s_{t},a_{t}\right)\leftarrow Q\left(s_{t},a_{t}\right)+\alpha \left[r_{t}+\gamma \max_{a^{\prime }}Q\left(s_{t+1},a^{\prime }\right)-Q\left(s_{t},a_{t}\right)\right]$$

where:

- $Q(s_{t},a_{t})$: estimated value of action $a_{t}$ in the current state $s_{t}$;
- $r_{t}$: reward received after executing the action at time *t*;
- $s_{t+1}$: next state;
- $\max_{a^{\prime }}Q\left(s_{t+1},a^{\prime }\right)$: highest-valued possible action in the next state;
- α: learning rate, which determines the weight of new and old Q-values;
- γ: discount factor, which determines the importance of future rewards.

Equation (21) describes how Q-Learning updates the Q-value by combining new information with old information. The UAVs repeat this process repeatedly, learning which action will provide the highest reward in each situation. The result of this learning process is that the UAVs have adopted routes and task allocations that not only reduce distance and time but also avoid collisions in a dynamic and uncertain environment. Thus, Q-Learning enables both real-time route optimisation and dynamic task allocation in MTSP, improving the overall performance of the swarm [117,128].

Q-Learning enhances the discrete decision making capabilities of UAVs, whereas DQN addresses large state spaces. The work in [128] proposes DQN-based trajectory planning for UAVs and observes better performance in complex urban settings. DQN combines the same principle with DQN to address high-dimensional state spaces, as demonstrated by the authors of [20] for human-level decision making.

Recent research has used Q-Learning and DQN for complex combinatorial optimisation problems such as UAV trajectory planning and the MTSP. For example, the study [118] used DQN in UAV swarms to improve real-time path selection and reduce mission completion time in dynamic scenarios. Similarly, the work [22] presented a Q-Learning-based task allocation approach for multi-UAV MTSP, which significantly improved load balancing among cooperative UAVs. Furthermore, the research presented in [129] employed a DQN-based approach for UAV collision avoidance and adaptive navigation, yielding promising results in complex environments.

#### 6.4.4. Actor–Critic Methods

**Figure 9:**
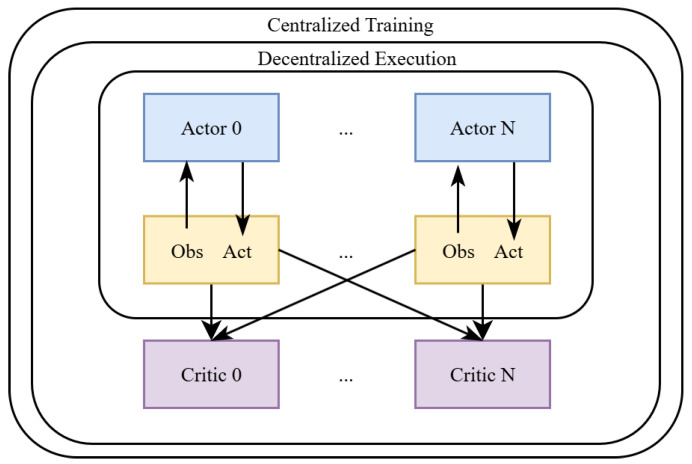
Representation of the Actor–Critic framework for UAV swarm trajectory optimisation.

Actor–Critic is one of the primary reinforcement learning methods that combines policy-based and value-based approaches [130,131]. These methods consist of two main parts, as illustrated in Figure 9:

Actor: which chooses an action and learns a policy π(a|s). Critic: which estimates the value of the selected action (V(s) or Q(s,a)) and provides feedback to the actor.

These methods are particularly suitable for problems where the action space is continuous, such as speed, angle, or throttle control, because they require precise and smooth control at each step [132]. In the initial scenario, UAVs must not only decide which path to take but also make smooth adjustments to speed and angle while following that path, so that mission time is short and energy use is efficient. In such cases, the Policy Gradient update rule is used, which adjusts the policy parameters in such a way that the expected total reward is maximised:

(22) $$\nabla_{\theta }J\left(\theta \right)=E_{\pi_{\theta }}\left[\nabla_{\theta }\log\pi_{\theta }\left(a|s\right)\cdot A\left(s,a\right)\right],$$

where:

- θ: policy parameters;
- $\pi_{\theta }\left(a|s\right)$: probability of choosing action *a* in state, *s*;
- A(s,a): advantage function, which expresses the utility of an action relative to the average.

Actor–critic methods have been used in UAV swarm research through several advanced implementations:

- Proximal Policy Optimisation (PPO): The article [132] introduced PPO, which is a stable and sample-efficient Actor–Critic algorithm. For UAVs, PPO-based frameworks have been successfully adopted for dynamic mission planning and MTSP coordination [115].
- Deep Deterministic Policy Gradient (DDPG): The authors of [133] proposed DDPG for continuous control. In UAV swarms, DDPG is utilised to learn continuous parameters, such as velocity and angle, resulting in smoother trajectories.
- Soft Actor–Critic (SAC): It is an Actor–Critic variant based on maximum entropy RL, which provides a better balance between exploration and exploitation. SAC has shown promising results in UAV collision avoidance and coverage scenarios [134].
- Hybrid Multi-Agent Actor–Critic Approaches: Huang et al. [135] used the Actor–Critic architecture in the multi-agent counterfactual advantage (MACA) framework, which reduced collisions in UAV swarms by 90% and improved cooperative behaviour.

Actor–critic methods enable UAV swarms to make adaptive decisions in complex and continuous action domains. In the context of problems such as MTSP, these approaches would allow UAVs to manage the trade-off between local observations and global mission objectives; however, they also present challenges in terms of computational complexity and scalability in large-scale swarms [117,131].

#### 6.4.5. Imitation Learning

**Figure 10:**
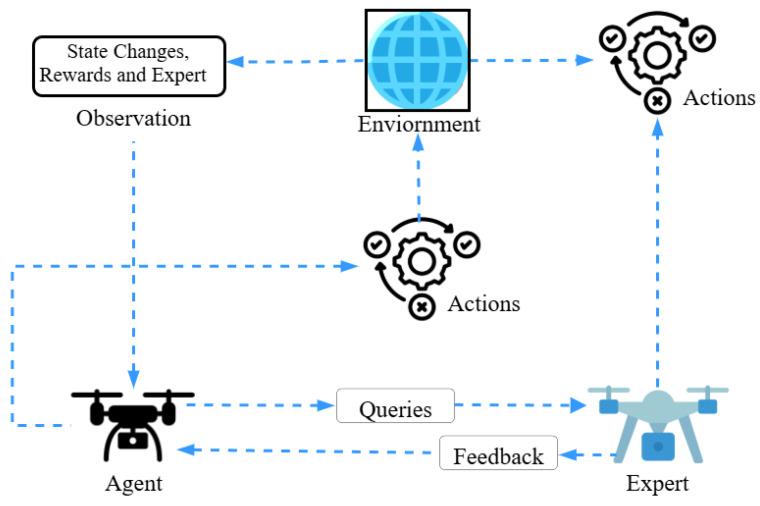
Representation of Imitation Learning in UAV trajectory planning.

Imitation Learning is a learning method based on the principle that a model learns to make better decisions by following the demonstrations of experts [119,136]. As depicted in Figure 10, it uses data provided by human operators or expert agents to learn a new policy that performs the same actions as the expert. This method is more efficient than reinforcement learning, because it learns from expert demonstrations rather than “trial-and-error”.

Imitation Learning is particularly effective in the context of UAV trajectory planning and the MTSP. For example, Kim et al. [137] employed an Imitation Learning framework for UAV swarms, enabling UAVs to replicate expert trajectories and enhance cooperative formation flying. Similarly, Wan et al. [138] proposed the DAgger (Dataset Aggregation) algorithm, which enhances learning robustness through iterative expert corrections in UAV navigation and decision making. Furthermore, Pan et al. [139] combined Imitation Learning with deep neural networks in UAV-based MTSP missions to significantly reduce planning time and increase mission efficiency. Imitation Learning approaches have been combined in multi-agent setups for UAV swarms, as in Zhang et al. [140], who developed a hybrid imitation–reinforcement learning framework that initialises UAVs with expert data and then further improves performance through reinforcement learning.

In behaviour cloning, the goal of the model is to replicate the behaviour of the expert with maximum accuracy. To achieve this goal, a specialised loss function is used, which measures the difference between the predicted action and the actual action of the expert. The mathematical expression for this loss is as follows:

(23) $$L\left(\theta \right)=\sum_{(s,a)\in D}{∥a-\pi_{\theta }\left(s\right)∥}^{2},$$

where:

- *D*: training dataset, consisting of pairs (s,a); where *s* is the state and *a* is the expert action;
- $\pi_{\theta }\left(s\right)$: action predicted by the policy network;
- *a*: actual action of the expert;
- $∥a-\pi_{\theta }{\left(s\right)∥}^{2}$: squared error between the prediction and the actual action.

This loss function teaches the policy to replicate the expert’s actions as accurately as possible. The higher this error, the greater the loss, and the model will reduce this difference by updating its parameters θ.

Imitation Learning not only enables UAVs to learn rapidly from expert demonstrations but also provides data-efficient and low-cost training for complex multi-target missions such as MTSP. However, expert data collection and domain shift can be a challenge in large-scale UAV swarms [119,136].

#### 6.4.6. Active Inference

**Figure 11:**
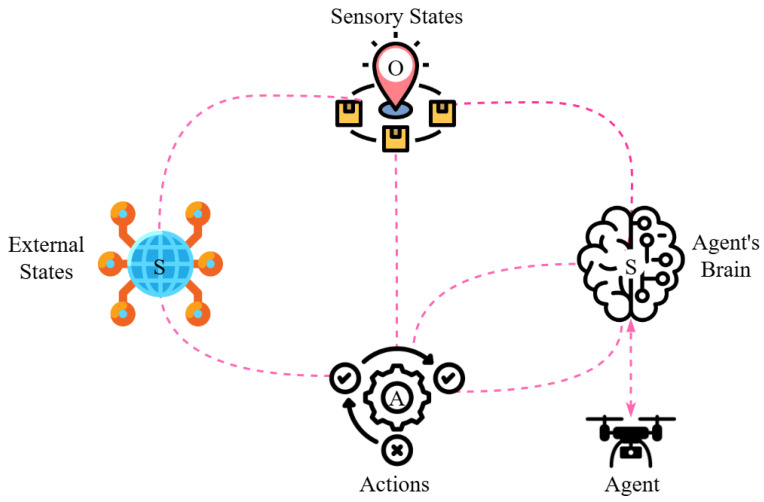
Representation of the Active Inference framework for UAV trajectory planning.

In Active Inference, decisions are based on the principles of free energy or surprise minimisation, which are inspired by theoretical models of the human brain and have been adapted to machines. It is an emerging probabilistic decision making framework based on Bayesian theory, integrating prediction, planning, and action under a unified framework [120,141]. In this approach, illustrated in Figure 11, the agent constructs a generative model that captures the world’s model. Through this model, the agent minimises the gap between the expectations of sensory input and the actual observations. This gap is called free energy, and minimising it allows the agent to make more adaptive decisions. This function measures the deviation between the agent’s belief and the model, expressed mathematically as:

(24) $$F=E_{q(s)}\left[\log q\left(s\right)-\log p\left(s,o\right)\right],$$

where:

- q(s): posterior belief of the agent about a state, *s*;
- p(s,o): generative model, which represents the joint probability of state, *s*, and observation, *o*.

This function forces UAVs to learn in such a way that the difference between their belief and the actual model is minimised.

In the context of MTSP, this method enables UAVs to design a trajectory and path based on predictions, thereby enhancing adaptation and facilitating real-time adjustments during the mission. As a result, UAV swarms reach their targets with greater precision and coordination, using minimal energy, regardless of the uncertain environment.

Applications of Active Inference have emerged in UAV research in recent years. For example, a goal-directed approach includes the TSPWP world model, which provides dictionary-based planning for effective flight by minimising surprises to a UAV in areas with wireless coverage. This model shows better results than Q-Learning in terms of decision making speed and stability, although further experiments are needed for full integration at the swarm level. [142]. In the same vein, Active-MGDBN (a hybrid of Gaussian Dynamic Bayesian Network) is introduced, which provides autonomous path planning and self-supervision, and increases flight flexibility and speed by suggesting optimal paths based on assumptions in an unknown network environment. At the same time, it does not require training on specific datasets, as it is capable of learning autonomously [25]. Another model is inspired by the decision-making style of human drivers, where decisions are made based on Bayesian cognition and free-energy minimisation. Although this model has not yet been directly applied to UAVs, its theoretical relevance makes it readily extensible to challenges such as UAV collision avoidance [143]. Smith et al. [144] showed that Active Inference enables UAVs to make successful decisions even in partially observable and dynamic environments. In contrast, Pezzulo et al. [145] provided predictive awareness to UAVs during missions through Bayesian Active Inference models. Furthermore, Millidge et al. [146] proposed a deep Active Inference framework that combines generative models with deep neural networks for UAVs, showing encouraging results in complex scenarios such as multi-target planning. Overall, Active Inference enable adaptive and prediction-driven decision-making capabilities to a UAV swarm. It provides a strong theoretical foundation through which UAVs can learn stable navigation in uncertain environments and effectively achieve speed, coordination, and continuously updated strategies during complex missions.

### 6.5. Challenges in AI-Based Algorithms

In the context of UAV swarms, various AI-based methods are employed to solve complex problems, such as the MTSP, effectively. Each algorithm solves a problem more effectively based on its learning style and neural processing; however, it also has some weaknesses. In recent years, several research works have demonstrated how one method succeeds another and overcomes its shortcomings, ultimately leading to the emergence of a generative and explainable short language model as a robust and unified framework [116,117,144].

Table 4 presents a comparison of the basic concepts and roles of different AI-based methods in UAV swarms and MTSP. It shows that each method is effective in specific situations but has its limitations; therefore, a combination of different AI techniques can be more flexible, scalable, and provide better results in uncertain environments.

This study begins with MARL, which is designed for multi-agent coordination and cooperative task allocation in UAV swarms [121,122,124]. MARL gave UAVs the ability to learn and cooperate; however, it still had problems such as scalability, communication overhead, and multi-agent credit assignment.

Then, came DRL, which is capable of learning whole mission-level policies [20,126]. However, DRL requires large amounts of data, time, and computational resources. This limitation is alleviated by value-based methods such as Q-Learning/DQN, which are effective for small and discrete action spaces. However, they are not suitable for continuous UAV control [128].

The next is Actor–Critic methods, which combine policy and value learning and are effective for continuous actions, such as speed and angle [132,133]. However, these methods can be unstable without hyper-parameter tuning. Imitation Learning took a step further, enabling UAVs to learn rapidly based on expert data [137,138]. However, when new or unforeseen situations arise, it demonstrates limited adaptability.

After addressing these problems, Active Inference emerged as a promising solution, based on Bayesian generative models that combine observation, prediction, and action into a unified framework [120,144]. Active Inference works effectively even with limited data, providing UAVs with adaptive decision-making capabilities in uncertain environments and enabling real-time mission execution.

Overall, this progressive evolution demonstrates how each approach addresses the weaknesses of the previous one, and ultimately, Active Inference emerges as a state-of-the-art, adaptable, and computationally efficient method for complex multi-agent problems, such as UAV swarm trajectory planning and MTSP.

Table 5 illustrates when and where different approaches are used to solve complex problems such as UAV trajectory planning and MTSP. While TAs are simple and computationally efficient, they are limited to static situations. BIAs are helpful for more complex and large-scale optimisation; however, they require parameter tuning and computational resources. AI-based approaches, particularly DRL and Active Inference, are most promising in high-dimensional and uncertain scenarios; however, they require specialised expertise, advanced computational setups, and often large training datasets.

### 6.6. Hybrid Methods

Efficient trajectory design for UAV networks can be achieved using hybrid techniques such as 2-OPT, genetic algorithms (GA), and Active Inference. Initially, the 2-OPT algorithm is employed to generate offline training examples, where UAV paths are optimised based on minimum distance and time. These data are then used to train a world model, enabling the UAV to self-supervise its environment and select an online policy through Active Inference [25]. Another study proposed a GA-based hybrid approach to generate repulsion forces in UAV swarm paths, thereby reducing collisions, overlaps, and interference among UAVs while producing optimal paths under the challenges of MTSP [147]. The data generated by 2-OPT are fed into an Active Inference model, allowing UAVs to analyse online situations, adapt their policies accordingly, and perform fast, stable, and reliable path planning. This hybrid framework enables UAVs not only to learn from offline training but also to make optimal decisions in real time through online Active Inference, resulting in significant improvements in network performance, overall capacity, and the sustainability of route planning [142].

## 7. Online and Offline Training and Testing: In the Context of UAV Swarms

Offline Training

The UAV swarm trajectory planning model is trained on previously collected data (trajectory sets, mission requirements, obstacle maps). This process is often conducted in a simulator or controlled environment to enable UAVs to learn effective policies before they are deployed on a mission. Once the model has completed training, it is deployed in the field [142,148].

Online Training

The model receives new observations in real time and continuously updates its policy. This method is essential in dynamic and uncertain environments because it enables UAVs to make adaptive decisions during the mission [142,149].

Table 6 illustrates a comparative overview of key aspects of offline and online learning in UAV swarms. The comparison reveals that offline training offers a safer and less complex approach, while also having room for improvement in terms of flexibility. In contrast, online training provides real-time adaptation, albeit at the expense of requiring more computational resources and increasing the risk of field errors.

### 7.1. Integration of Offline Training with Online Testing

An effective strategy for UAV swarm missions is to utilise offline training for initial learning, followed by validation and fine-tuning of the model in the field through online testing.

#### 7.1.1. Offline Phase: BIA’s Generated Data with Supervised/Unsupervised Learning

In the offline phase, the goal is to train an AI policy using data generated from BIAs (such as GA, PSO, or ACO) to learn expert-level performance [150]. To achieve this, a dataset definition is first defined, which consists of states and their corresponding actions:

(25) $$D={\left\{\left(s_{i},a_{i}\right)\right\}}_{i=1}^{N},$$

where *D* is the trajectory plan generated by the BIAs, and each pair $\left(s_{i},a_{i}\right)$ represents a particular state and its corresponding expert action.

Based on this dataset, a loss function is defined so that the AI policy $\pi_{\theta }\left(s\right)$ can accurately replicate the expert’s actions. The following optimisation problem is solved to minimise this loss:

(26) $$\theta^{*}=\arg\min_{\theta }\sum_{(s,a)\in D}{∥a-\pi_{\theta }\left(s\right)∥}^{2},$$

where:

- *D*: dataset generated by algorithms such as GA, PSO, or ACO;
- *s*: state of the environment (e.g., location of the UAV or remaining targets);
- *a*: action (trajectory segment or assignment) recommended by the BIAs;
- $\pi_{\theta }\left(s\right)$: AI-based policy that is learning to predict actions for these states.

Through this process, the AI policy can learn from the expert algorithm’s decisions to enhance path planning and task allocation in the MTSP, enabling effective and autonomous decision making without requiring expert assistance in the future.

#### 7.1.2. Online Phase (AI-Based Fine-Tuning)

During the online phase, the model learns from the environment in real time to further refine the policy it has previously learned. This process is achieved through RL-based fine-tuning, where the UAV updates its policy based on its observations and the rewards it receives, thereby improving mission performance [151]. The following policy update equation is used for this purpose:

(27) $$\theta_{t+1}=\theta_{t}+\alpha \cdot \nabla_{\theta }\log\pi_{\theta }\left(a_{t}|s_{t}\right)\cdot r_{t},$$

where:

- θ: parameters of the AI model, which are updated during the learning process;
- $s_{t},a_{t}$: current state and currently selected action;
- $r_{t}$: reward received after the action, which reflects the effectiveness of the action;
- α: learning rate, which determines how much impact each update will have.

Equation (27) ensures that the UAV updates its policy toward actions with higher expected rewards.

In MTSP scenarios, this update mechanism enables real-time path and speed optimisation, rapid adaptation to new targets, and improved inter-UAV coordination, enhancing overall mission success.

In this approach, BIAs (e.g., GA, PSO, ACO) are employed to generate initial datasets and trajectories, which subsequently serve as training inputs for AI-based models. This dual strategy not only provides UAV swarms with a robust initial policy but also enables them to perform adaptive decision making in real time, significantly increasing both mission success and safety [150,151,152].

## 8. Decision Making and Collision Avoidance in UAV Swarms

### 8.1. Decision Making in Swarms

Decision making is a fundamental challenge in UAV swarm systems, as each UAV must not only focus on its mission (such as task execution or trajectory following) but also make real-time decisions while cooperating with other UAVs. The accuracy of these decisions is critical for mission success, collision avoidance, efficient energy use, and overall system stability [153,154].

Decision making is typically described at two levels: Local decision making, where each UAV makes decisions based on its local information (such as sensor data and the positions of nearby UAVs). Collective decision making, where UAVs share data and act according to a global strategy [155].

### 8.2. Online and Offline Decision Making

Offline decision making: In offline decision making, UAVs rely on pre-trained policies or role-based models, which are often trained on simulations or historical data. This approach is computationally lightweight and suitable for predictable missions (such as mapping or fixed survey paths) [156].

Online decision making: Online decision making is more dynamic, where UAVs continuously observe the environment, share information, and make decisions in real time based on the current situation (such as sudden obstacles, changing weather conditions, or a new mission target). This approach makes UAV swarms more adaptive and resilient, but it requires more computational power and a robust communication structure [154].

Modern research is moving in the direction of using both methods in a hybrid manner, that is, first providing UAVs with a basic decision policy through offline learning and then continuously improving it through online decision making during the mission [152,157].

Table 7 illustrates a comparison of offline and online decision making in UAV swarms, showing that offline methods have low computational demands and rely on pre-trained policies. In contrast, online decision making offers greater adaptability and flexibility in real time, but requires more computational resources.

### 8.3. A Challenge in Decision Making: Collision Avoidance

When multiple UAVs fly together on close or shared paths, the risk of collision increases. This is a fundamental challenge for UAV swarms, as a minor collision can not only damage one UAV but also fail the entire mission. Therefore, collision avoidance strategies are considered an integral part of decision making. Modern research has shown that various approaches are used to improve collision avoidance in UAV swarms, including geometric, potential field-based, optimisation-driven and AI-assisted methods [158,159].

Collision avoidance: Techniques by which UAVs avoid each other or obstacles to maintain mission safety. Decision making in UAV swarms is not limited to path selection, but is a continuous, informative and protective process, involving real-time perception and mutual coordination. Especially in dynamic and uncertain environments, online decision making and collision avoidance are inseparable [160].

### 8.4. Modern and Scientific Methods for Collision Avoidance

Several approaches have been developed for collision avoidance in UAV swarms, which can be categorised into the following groups.

#### 8.4.1. Geometric Methods:

These methods are based on the geometry of the velocity and position of UAVs. For example, in the velocity obstacle method (VOM), each UAV predicts its future position based on the current position and velocity of other UAVs, and adjusts its velocity to avoid potential collisions [161]. The basic concept in this method is to define a velocity obstacle set, which is the set of all velocity vectors that could lead to a collision in the future. This set can be expressed mathematically as:

(28) $$VO_{i|j}=\left\{v_{i}\;|\;\exists \;t>0:\;p_{i}+v_{i}t=p_{j}+v_{j}t\right\},$$

where:

- $VO_{i|j}$ is the set of all possible velocities of UAV, *i*, that can cause a collision with UAV *j*.
- $p_{i},p_{j}$ are the current positions of UAV, *i*, and UAV *j*.
- $v_{i},v_{j}$ are the current velocities of UAV, *i*, and UAV *j*.
- *t* is the time in the future when the collision can occur.

If $v_{i}$ is part of this set, UAV, *i*, can collide with UAV *j* in the future. In this case, UAV, *i*, should change its velocity and adopt a safe alternative vector. In the context of MTSP, Equation (28) enables UAVs to not only optimise their routes in real time but also complete missions at a safe distance from each other, regardless of the proximity of their routes. This helps to avoid collisions, reduce mission completion time, and improve team coordination.

#### 8.4.2. Force Field Approaches

Potential field-based approaches to UAV navigation are based on the concept that the mission target and obstacles in the environment produce attractive and repulsive forces, respectively. These forces can be expressed mathematically as a total force function, which determines the direction and magnitude of the UAV’s motion.

(29) $$F=F_{attract}+F_{repel},$$

where:

- $F_{attract}$: force that attracts the UAV towards the target;
- $F_{repel}$: force that repels the UAV from the obstacles.

These two forces together enable the UAV to take a smooth and safe path, where the attraction force encourages it to reach the target while the repulsion force ensures collision avoidance [162,163,164].

In the context of MTSP, this function not only provides UAVs with an effective path to the target but also helps to avoid collisions and reduce mission completion time in multi-UAV operations.

#### 8.4.3. Optimization-Based Methods

Optimisation-based collision avoidance approaches are based on the principle that each UAV should choose its path in such a way that the overall mission cost is minimised, while also meeting the requirements for collision avoidance. For this, a cost function is defined that incorporates both mission performance and safety conditions.

(30) $$\min_{x}\;J\left(x\right)\;s.t.\;\left|x_{i}-x_{j}\right|\geq d_{safe},\;\forall i\neq j,$$

where:

- J(x): overall mission cost (e.g., time, distance, or energy);
- $x_{i},x_{j}$: positions of UAV, *i*, and UAV *j*;
- $d_{safe}$: minimum safe distance that must be maintained between UAVs.

This optimisation problem ensures that each UAV updates its path in a way that not only completes the mission at the lowest cost but also stays at a safe distance from other UAVs.

In the context of MTSP, this method is particularly effective in multiobjective scenarios, as UAVs can simultaneously improve both mission efficiency and flight safety [165].

#### 8.4.4. Lennard–Jones Potential

The Lennard–Jones Potential model is a physical model that describes the balance of attractive and repulsive forces between two UAVs [166]. This concept is utilised in collision avoidance algorithms to prevent UAVs from becoming too close or too far apart. The following potential function mathematically represents this model:

(31) $$U\left(d\right)=\epsilon \left[{\left(\frac{\sigma }{d}\right)}^{12}-2{\left(\frac{\sigma }{r}\right)}^{6}\right],$$

where:

- *d*: distance between the two UAVs;
- ϵ: parameter controlling the magnitude of the potential;
- σ: distance at which the potential is at its minimum value.

The Lennard–Jones model generates strong repulsion at close range and weak attraction at intermediate range, allowing UAVs to maintain a safe distance and avoid collisions [167].

In the context of MTSP, the Lennard–Jones Potential enables UAVs to adopt a balanced behaviour, efficiently completing their paths while maintaining coordination within the swarm, especially in narrow or complex mission areas.

#### 8.4.5. Harmonic Potential

Harmonic potential is an effective mathematical model that imposes a penalty for deviations from the desired distance between two UAVs or between a UAV and a target. The basic concept relies on a quadratic function, where energy or potential increases with deviation [168]. This function ensures that the UAVs remain within the desired distance $d_{0}$. Mathematically, it is expressed as:

(32) $$U\left(d\right)=\frac{1}{2}k{\left(d-d_{0}\right)}^{2},$$

where:

- *d*: current distance;
- $d_{0}$: desired or target distance;
- *k*: spring constant, which controls the magnitude of the correction.

This provides a soft corrective mechanism, as minor deviations incur a small penalty, while large deviations incur a significantly larger penalty.

In the context of MTSP, the harmonic potential is beneficial for formation-based missions, where UAVs must reach targets while maintaining a certain distance. The harmonic potential method not only ensures collision avoidance but also improves swarm coordination and mission performance.

#### 8.4.6. Gaussian Repulsion Force

The Gaussian repulsion force is designed to generate a repulsive force as the distance decreases. Still, this force increases or decreases smoothly so that there are no sudden changes in the movement. This has the advantage that the movement of the UAVs remains more natural and stable, especially when the swarm formation is dense [169,170]. Mathematically, this potential function is expressed as:

(33) $$U\left(d\right)=A\cdot \exp\left(-\frac{{\left(d-\mu \right)}^{2}}{2\sigma^{2}}\right),$$

where:

- *d*: current distance between the two UAVs;
- *A*: maximum amplitude of repulsion;
- μ: distance around which repulsion is most effective;
- σ: spread parameter, which determines the extent of the repulsion effect.

This Gaussian repulsion force method prevents sudden changes in motion or direction, allowing UAVs to move in a smooth and coordinated manner. In the context of MTSP, the Gaussian repulsion force protects UAVs from collisions in dense aerial scenarios, while also ensuring stable swarm alignment and improved mission performance.

#### 8.4.7. Inverse Quadrature/Artificial Potential Field (APF)

In the APF method, the mission target generates an attractive force while obstacles generate a repulsive force. This repulsive force is designed to keep the UAV away from obstacles, thereby avoiding collisions. This model utilises a potential function based on the inverse square of the distance from the obstacle, which increases rapidly as the obstacle is approached. Mathematically, the repulsive potential can be described as:

(34) $$U_{rep}\left(d\right)=\frac{1}{{\left(d-d_{0}\right)}^{2}},$$

where:

- *d*: current distance between the UAV and the obstacle;
- $d_{0}$: safe or minimum allowed distance.

As *d* approaches $d_{0}$, the collision potential increases significantly, forcing the UAV to change direction and ensuring collision avoidance [171].

In the context of MTSP, the APF method enables UAVs to navigate towards the target while avoiding obstacles, thereby accelerating mission completion and maintaining swarm coordination.

#### 8.4.8. Priority-Based Strategies

In certain scenarios, UAVs employ simple rule-based strategies to avoid collisions or manage air traffic. For example, a UAV may slow down or stop to let another UAV pass first. This strategy does not rely on complex mathematical models or heavy computational processing, making it a low-computational heuristic that is particularly effective in congested airspace [172].

In the context of MTSP, priority-based strategies enhance inter-UAV route coordination, minimise unnecessary interference, and reduce mission completion time. It is especially beneficial when airspace is limited or swarms have to operate nearby.

### 8.5. Challenges in Collision-Avoidance Methods

Many of these approaches, geometric, force field, optimisation-based, and heuristic, offer distinct advantages; however, they also have some drawbacks.

- Geometric methods: These are mathematically simple and fast in real time. However, they can be less flexible in dynamic and uncertain environments, and their accuracy may suffer in complex scenarios.
- Force field methods: These provide smooth and safe paths. However, they can become stuck in local minima and are ineffective in environments with complex constraints.
- Optimisation-based methods: improve performance and safety simultaneously. Nevertheless, they have high computational cost and can be slow in large swarms or real-time applications.
- Heuristic methods: are simple, fast, and require fewer computational resources. However, they do not always provide the best solution and may fail in complex or unpredictable situations.

The current trend is towards developing hybrid systems that integrate physics-based and AI-driven collision-avoidance techniques. This combination can significantly improve the reliability, adaptability, and mission performance of UAV swarms by combining the strengths of each method [151,160,173].

Table 8 illustrates various collision-avoidance methods. Each method has distinct advantages and limitations, and hybrid approaches are often employed for more effective results in practical scenarios.

**Table 8:** Different collision-avoidance methods in UAV swarms and their applications.

Method	Explanation	Role in UAV Swarms
Geometric	Geometry-based analysis of velocity and path, such as velocity obstacle (VO) or reciprocal velocity obstacle (RVO).	Fast and computationally light; effective in low-density swarms and predictable environments [161].
Force field	Combination of attractive and repulsive forces; target pulls and obstacle pushes.	Generates intuitive and smooth paths, but can become stuck in local minima [162,163].
Optimisation	Minimises the objective function with collision avoidance constraints.	Very effective in multi-UAV coordination, but computationally demanding [165].
Lennard–Jones	Physics-inspired potential that provides short-range repulsion and medium-range attraction.	Useful for formation flights and maintaining safe separation [167].
Harmonic potential	Quadratic potential that provides a penalty on deviation.	Helpful in information-keeping and smooth trajectory generation [168].
Gaussian repulsion	Gaussian-based repulsive field that increases in intensity with distance.	Provides soft yet strong repulsion and reduces sudden manoeuvres [169].
Inverted quadrant/APF	Classical artificial potential field model: target is attractive, and obstacles have repulsive potential.	Easy implementation; but the problem of local minima remains [171].
Waiting or yielding rules	Priority-based heuristics: UAV stops or slows down to let other UAVs go first.	Simple and effective in decentralised systems; basic safety measure in congested airspaces [172].

## 9. Challenges in UAV Swarm Trajectory Planning

Trajectory planning for UAV swarms is a complex, multifaceted problem with numerous challenges. The most fundamental challenge is to determine safe, energy-efficient, and collision-free trajectories in real time in a dynamic and unpredictable environment. Collision avoidance and effective coordination among multiple UAVs, particularly in the presence of limited communication resources and latency, are key issues. In addition, the presence of obstacles, deceptive signals, and weather uncertainties also affects the accuracy of trajectory planning.

### 9.1. Explainability

Most modern algorithms, including deep learning and reinforcement learning, operate as black-box models whose internal logic is not readily explainable. Making the decision-making process transparent in UAV swarm systems is an indispensable requirement for maintaining trust and auditability within the system.

### 9.2. Online Learning

Effective decision making in a rapidly changing environment requires online learning capabilities, which are currently limited or unstable in existing models. Keeping models that are updated online stable without overfitting is a significant challenge.

### 9.3. Lack of Incremental Learning

Modern automated systems should improve themselves by learning from the environment, but models often lose previous information while learning new data. To overcome this challenge, incremental learning methods that maintain the continuity of information are necessary.

### 9.4. Energy Efficiency

UAVs have limited battery life, and more complex algorithms or frequent path retracing increase energy consumption. Effective trajectory planning must also consider energy efficiency.

### 9.5. Security and Network Protection

UAVs are susceptible to network-based and communication attacks. Attacks such as enemy signals, GPS spoofing, or data poisoning during trajectory planning can paralyse the system. Current models have poor defences against these threats.

### 9.6. Coordination and Coordination

Avoiding collisions between multiple UAVs and enabling efficient distributed performance is a constant challenge, especially when each UAV is making decisions autonomously and there is no central system.

### 9.7. Dynamic Environment Adaptation

Current models often operate in static or semi-dynamic environments, but in the real world, threats, obstacles, or user preferences are constantly changing. Rapid and accurate decision making under such conditions is limited in current systems.

### 9.8. Scalability and Real-Time Performance

Algorithms that have proven successful on small systems often fail when tested on large numbers of UAVs. Lack of scalable design can be a barrier to large-scale missions.

### 9.9. Sensor Limitations and Noisy Observations

UAVs often rely on sensors of poor quality or high noise levels, which can lead to suboptimal trajectory planning or unsafe flight paths. Current algorithms have a limited ability to perform noise-hardened decision making.

### 9.10. Lack of Standardised Evaluation Metrics

There is still no internationally recognised uniform metric for trajectory planning, making it difficult to compare different models scientifically. This deficiency is hindering research progress.

## 10. Future Research Directions

Given the current challenges in UAV swarm trajectory planning, future research should prioritise technical directions that deliver effective and flexible solutions while incorporating explainability, online learning, and principles inspired by biological motion.

### 10.1. Data Generation from BIAs

The lack of training datasets poses a significant challenge, particularly for RL or Active Inference models. To address this issue, diverse and realistic synthetic trajectories can be generated using BIAs such as GA, PSO, or ACO. This data can not only be used for pre-training but can also be used to power other models through transfer learning.

### 10.2. Active Inference as a Comprehensive Solution

Active Inference is a modern and emerging framework based on Bayesian brain theory. This model unifies perception, decision making, and learning simultaneously. In the context of UAVs, Active Inference not only enables decision making under uncertain conditions but also maintains the internal transparency of the system. The most prominent feature of this model is that it self-learns policies to minimise prediction error, making it particularly suitable for dynamic and partially observable environments.

### 10.3. Explainable Reinforcement Learning (XRL)

Although traditional RL models possess strong learning abilities, their decisions are often opaque and difficult to understand. Explainable RL techniques such as attention-based models, saliency maps, or policy summarisation will be crucial for the reliability and human-in-the-loop validation of UAV systems in the future. It will enhance confidence in decision making, particularly in both civilian and military applications.

### 10.4. Integration of Online and Incremental Learning

TAs often rely on offline training, which can be ineffective in practical applications where the environment changes over time. In the future, lightweight and real-time adaptable models are needed that can immediately learn from new observations and improve their policy based on online learning. Approaches such as Active Inference, meta-learning and continual learning frameworks can be effective in this context.

### 10.5. Energy-Efficient and Scalable Models

Given the limited battery and computational resources in UAVs, there is a need for computationally lightweight models that consume low power and can efficiently run on microcontrollers or embedded systems. Neuromorphic computing, spiking neural networks, and edge AI solutions can play a crucial role in this direction.

### 10.6. Integrated Frameworks

A comprehensive framework is needed in the future that integrates all aspects, such as multi-agent coordination, real-time adaptation, explainability, and safety into a unified structure. For this purpose, approaches such as Active Inference or Hybrid Decision Systems with hierarchical reinforcement learning are promising.

## 11. Conclusions

This review presents a comprehensive, comparative, and critical analysis of the latest trends and techniques in trajectory planning for UAV swarms. The study highlights three main algorithmic streams: traditional algorithms, biologically inspired algorithms, metaheuristics, and artificial-intelligence-based strategies. It demonstrates how these approaches, along with their respective strengths and limitations, influence the design of trajectories in UAV swarm missions. The study reveals that TAs offer an excellent foundation for structured and static environments, but are limited in dynamic and uncertain situations. BIAs have achieved remarkable success in global optimisation and solution diversity; however, challenges such as convergence speed and computational load persist. Finally, AI-based strategies, particularly DRL, MARL, and Active Inference, have opened up new possibilities for adaptive decision making, decentralised control, and real-time trajectory adjustments in UAV swarms. However, these approaches also face challenges such as computational complexity and data dependency. Furthermore, the research indicates that hybrid frameworks, combining the reliability of TAs, the global search capability of BIAs, and the adaptability of AI techniques, offer promising future solutions for UAV swarm trajectory planning. These hybrid approaches establish a compelling exploration–exploitation balance in multi-agent missions and have the potential to address key challenges such as collision avoidance, scalability, and mission efficiency.

This work identifies several critical areas for future research, including the need for real-time and online learning frameworks that enable UAV swarms to rapidly adapt to new scenarios during missions. Similarly, the integration of explainable artificial intelligence (Explainable AI) and interpretable reinforcement learning (Interpretable RL) techniques is crucial for making decision-making processes more transparent and reliable. Furthermore, the development of hybrid bio-AI models for practical use in data- and resource-constrained environments is an important research direction. Ultimately, improvements in Simulation-to-Reality (Sim2Real) transfer methods will ensure that laboratory-trained models can be seamlessly deployed in real-world UAV missions.

## Figures and Tables

**Table 1:** A comparative review of path planning and trajectory design.

Aspect	Path Planning	Trajectory Designing
Goal	Minimum safe path	Smooth and timely path
Time included?	No	Yes
Speed/acceleration included?	No	Yes
Place of use	Map navigation	Autonomous flight, drones, robotics
Nature of environment	Static	Dynamic/uncertain
Complexity	Low	High

**Table 2:** Role of TAs in MTSP with comparison.

Algorithm	Advantages and Limitations	Role in MTSP	Time to Complete a Mission	Scalability	Energy Efficiency	Collision Rates
Dijkstra-based Algorithms	Efficient in static settings, though limited to a single UAV.	Path for each UAV.	Fast for static; moderate in dynamic.	Poor scalability.	Moderate.	Low in static; increases in dynamic.
A* Algorithm	Faster than Dijkstra, not for dynamic environments.	Heuristic guidance for movement.	Optimal for small swarms; moderate in large tasks.	Limited scalability.	High in static.	Low in controlled; moderate in dynamic.
RRT-based Algorithms	Fast search, effective in dynamic, non-smooth paths.	Obstacle avoidance in dynamic spaces.	Variable, slower in complex areas.	High scalability in a dynamic environment.	Low due to randomness.	Moderate, depends on smoothness.
DWA	Real-time planning, needs a global planner for large swarms.	Collision avoidance in cluttered spaces.	Quick for short-term plans.	Moderate with integration.	Moderate to high.	Low in real time.
Dubin’s Path	Effective for fixed-wing, not dynamic replanning.	Fulfilment of physical constraints.	Fast for fixed, not dynamic.	Low scalability.	High for fixed-wing.	Low if constraints met.

**Table 3:** Role of BIAs in UAV swarm and comparison.

Algorithm	Advantages and Limitations	Role in MTSP	Time to Complete a Mission	Scalability	Energy Efficiency	Collision Rates
PIO	Simple, effective for basic navigation; limited adaptability.	GPS-like orientation.	Moderate, struggles with complexity.	Low in dynamic tasks.	Moderate.	Low in controlled settings.
SSA	Easy to implement; poor in dynamic environments.	Leader–follower coordination.	Moderate, slow in complex tasks.	Moderate for small swarms.	Low–moderate.	Low in simple environments, higher in dynamic.
ABC	Balanced exploration and exploitation; slow convergence.	Workers find solutions and share info.	Slow due to convergence.	Moderate in large spaces.	Moderate.	Moderate, varies with exploration.
ACO	Effective pathfinding; suffers from pheromone imbalance.	Collective memory via pheromones.	Moderate, slow in large environments.	High in dynamic tasks.	High due to pheromone updates.	Moderate, depends on pheromone strength.
PSO	Fast convergence; can stagnate in local optima.	Fast, optimal solutions.	Fast for continuous tasks.	High in continuous optimisation.	High in controlled settings.	Low, may increase with stagnation.
GA	High diversity; slow convergence, high computational cost.	Global solutions via mutation and crossover.	Moderate, slow in complex spaces.	High in large solution spaces.	Moderate.	Low with maintained diversity.

**Table 4:** Using AI-based methods in UAV swarm and comparison.

Method	Advantages and Limitations	Role and Challenges in UAV Swarm	Time to Complete a Set Mission	Scalability	Energy Efficiency	Collision Rates
MARL	Cooperative. Poor scalability.	Learns policies, but scalability is challenging.	Moderate, depends on coordination.	Moderate for large swarms.	Moderate.	Moderate, complexity impacts coordination.
Deep RL	Full learning. High data and training costs.	Learns complex tasks, but slow due to data needs.	Slow due to training.	Low scalability for dynamic tasks.	High computational cost.	Low, but increases in dynamic settings.
Q-Learning/DQN	Fast for small tasks. Struggles with large spaces.	Effective in small spaces, but poor in complex ones.	Fast for small, discrete tasks.	Poor for continuous spaces.	Moderate.	High in small tasks, higher in complex ones.
Actor–Critic	Continuous control. Unstable without tuning.	Effective for continuous tasks but needs tuning.	Moderate, depends on tuning.	Moderate for complex tasks.	Moderate.	Moderate, instability increases risk.
Imitation Learning	Fast learning. Poor generalisation.	Learns fast, struggles with new scenarios.	Fast, but weak in new environments.	Low scalability in dynamic tasks.	Low energy.	High in controlled, low in new environments.
Active Inference	Adapts with limited data. Needs robust models.	Adapts well with minimal data, but needs efficient models.	Fast with minimal data.	High scalability with tuning.	High due to computational cost.	Low, adaptive nature helps avoid collisions.

**Table 5:** Comparison of different methods for trajectory planning review.

Method	Examples	Scope of Use	Skill Requirement
TA	Dijkstra, A*, RRT, DWA, Dubin’s Path	Known or static environments, pathfinding, and trajectory planning for individual UAVs or fixed-wing UAVs	Beginner to Intermediate
BIA	PIO, SSA, ABC, ACO, PSO, GA	Complex or large search spaces, swarm coordination, and optimisation in UAV swarms	Intermediate
AI-A	MARL, Deep RL, Q-Learning, Actor–Critic, Imitation Learning, Active Inference	Dynamic, stochastic, and uncertain environments, multi-agent cooperation, learning from experience	Advanced

**Table 6:** Comparative aspects of offline and online learning in UAV swarm.

Aspects	Offline Training	Online Training
Learning time	Before mission	During mission
Data source	Pre-existing data	Real-time field data
Computational complexity	Low	High
Model flexibility	Limited	High (adaptive)
Risk	Low (safe environment)	High (potential for error in field)

**Table 7:** Comparison of offline and online decision making in UAV swarms.

Aspect	Offline Decision Making	Online Decision Making
Decision time during mission	Pre-trained	Real-time
Adaptation	Static	Dynamic
Example	Pre-trained policy, static path	Real-time obstacle avoidance, task redistribution
Computational demand	Low	High

## Data Availability

The data presented in this study are available on request from the corresponding author.

## Abbreviations

The following abbreviations are used in this manuscript:

AI	Artificial Intelligence; Machine Intelligence; Cognitive Computing
TSP	Travelling Salesman Problem; Route Optimization Problem
MTSP	Multiple Travelling Salesman Problem; Multi-UAV TSP
TA	Traditional Algorithms; Classical Algorithms; Heuristic Algorithms
BIA	Biologically Inspired Algorithms; Evolutionary Algorithms
AI-A	Modern AI-based Algorithms; Advanced AI Methods
RL	Reinforcement Learning; Trial-and-Error Learning
MARL	Multi-Agent Reinforcement Learning;
PPO	Proximal Policy Optimization; Policy Gradient Methods
RRT	Rapidly-Exploring Random Trees
DWA	Dynamic Window Approach
ORCA	Optimal Reciprocal Collision Avoidance; Collision Avoidance
JPS	Jump Point Search; Optimized A* Search
PIO	Pigeon Inspired Optimization; Pigeon Navigation Algorithm
SSA	Salp Swarm Algorithm; Swarm Intelligence-based Algorithm
ABC	Artificial Bee Colony; Bee-based Algorithm
ACO	Ant Colony Optimization; Ant-inspired Search Algorithm
PSO	Particle Swarm Optimization; Swarm Intelligence Algorithm
GA	Genetic Algorithm; Evolutionary Algorithm
DRL	Deep Reinforcement Learning; Neural Network-based RL
DQN	Deep Q-Network; Q-Learning with Deep Networks
CTDE	Centralised Training and Decentralised Execution
DDPG	Deep Deterministic Policy Gradient
SAC	Soft Actor–Critic
MACA	Multi-Agent Counterfactual Advantage
APF	Artificial Potential Field
XRL	Explainable Reinforcement Learning

## References

[1] Ahmad F., Mirza M.Y., Hussain I., Arshid K. (2025). A Multi-Ray Channel Modelling Approach to Enhance UAV Communications in Networked Airspace. Inventions.

[2] Guan S., Zhu Z., Wang G. (2022). A Review on UAV-Based Remote Sensing Technologies for Construction and Civil Applications. Drones.

[3] Alqudsi Y., Makaraci M. (2025). UAV Swarms: Research, Challenges, and Future Directions. J. Eng. Appl. Sci..

[4] Ekechi C.C., Elfouly T., Alouani A., Khattab T. (2025). A Survey on UAV Control with Multi-Agent Reinforcement Learning. Drones.

[5] Shukla P., Shukla S., Singh A.K. (2024). Trajectory-Prediction Techniques for Unmanned Aerial Vehicles (UAVs): A Comprehensive Survey. IEEE Commun. Surv. Tutor..

[6] Gupta L., Jain R., Vaszkun G. (2016). Survey of important issues in UAV communication networks. IEEE Commun. Surv. Tutor..

[7] Dhulkefl E., Durdu A., Terzioğlu H. (2020). Dijkstra algorithm using UAV path planning. Konya J. Eng. Sci..

[8] Zhang L., Zhao M. (2021). Grid-based A* algorithm for UAV swarm scheduling in urban environments. J. Intell. Robot. Syst..

[9] Li L., Zhang F., Yu J., Zhu Q., Lu H., Liu S. (2023). Exact and Heuristic Multi-Robot Dubins Coverage Path Planning for Known Environments. Sensors.

[10] Gao W., Li Y. (2021). Search-based algorithms for UAV path planning: A comprehensive review. Appl. Sci..

[11] Kumar P., Pal K., Govil M. (2025). Comprehensive Review of Path Planning Techniques for Unmanned Aerial Vehicles (UAVs). ACM Comput. Surv..

[12] Tang R., Tang J., Talip M.S., Aridas N.K., Xu X. (2025). Enhanced Multi Agent Coordination Algorithm for Drone Swarm Patrolling in Durian Orchards. Sci. Rep..

[13] Shin J.J., Bang H. (2020). UAV path planning under dynamic threats using an improved PSO algorithm. Int. J. Aerosp. Eng..

[14] Alabbadi A.J., Sababha B.H. (2025). On the Optimization of UAV Swarm Aco-Based Path Planning. Jordanian J. Comput. Inf. Technol..

[15] Wang F., Xu G., Wang M. (2023). An improved genetic algorithm for constrained optimization problems. IEEE Access.

[16] Akay B., Karaboga D. (2009). A modified Artificial Bee Colony algorithm for real-parameter optimization. Inf. Sci..

[17] Awadallah M.A., Makhadmeh S.N., Al-Betar M.A., Dalbah L.M., Al-Redhaei A., Kouka S., Enshassi O.S. (2025). Multi-objective Ant Colony Optimization. Arch. Comput. Methods Eng..

[18] Chandan R.R., Soni S., Raj A., Veeraiah V., Dhabliya D., Pramanik S., Gupta A. (2023). Genetic algorithm and machine learning. Advanced Bioinspiration Methods for Healthcare Standards, Policies, and Reform.

[19] Pan Y., Yang Y., Li W. (2021). A deep learning trained by genetic algorithm to improve the efficiency of path planning for data collection with multi-UAV. IEEE Access.

[20] Mnih V., Kavukcuoglu K., Silver D., Rusu A.A., Veness J., Bellemare M.G., Graves A., Riedmiller M., Fidjeland A.K., Ostrovski G. (2015). Human-level control through deep reinforcement learning. Nature.

[21] Kaliappan V., Nguyen T., Jeon S., Lee J., Min D. Deep Multi Agent Reinforcement Learning Based Decentralized Swarm UAV Control Framework for Persistent Surveillance. Proceedings of the Asia-Pacific International Symposium on Aerospace Technology.

[22] Nguyen T., Pham H., Le M. (2021). Task allocation and trajectory optimization for UAV swarms via deep reinforcement learning. IEEE Access.

[23] Nguyen V.D., Yang Z., Buckley C.L., Ororbia A. (2024). R-aif: Solving Sparse-Reward Robotic Tasks from Pixels with Active Inference and World Models. arXiv.

[24] Krayani A., Alam A.S., Marcenaro L., Nallanathan A., Regazzoni C. (2022). A novel resource allocation for anti-jamming in cognitive-UAVs: An Active Inference approach. IEEE Commun. Lett..

[25] Krayani A., Khan K., Marcenaro L., Marchese M., Regazzoni C. (2024). Self-Supervised Path Planning in UAV-Aided Wireless Networks Based on Active Inference. Proceedings of the ICASSP 2024-2024 IEEE International Conference on Acoustics, Speech and Signal Processing (ICASSP).

[26] Page M., McKenzie J., Bossuyt P., Boutron I., Hoffmann T., Mulrow C., Shamseer L., Tetzlaff J., Akl E., Brennan S. (2021). The PRISMA 2020 Statement: An Updated Guideline for Reporting Systematic Reviews. BMJ.

[27] Yang Y., Xiong X., Yan Y. (2023). UAV formation trajectory planning algorithms: A review. Drones.

[28] Puente-Castro A., Rivero D., Pazos A., Fernandez-Blanco E. (2022). A review of artificial intelligence applied to path planning in UAV swarms. Neural Comput. Appl..

[29] Saeed R.A., Omri M., Abdel-Khalek S., Ali E.S., Alotaibi M.F. (2022). Optimal path planning for drones based on swarm intelligence algorithm. Neural Comput. Appl..

[30] Wang L., Huang W., Li H., Li W., Chen J., Wu W. (2024). A review of collaborative trajectory planning for multiple unmanned aerial vehicles. Processes.

[31] Sharma A., Shoval S., Sharma A., Pandey J.K. (2022). Path planning for multiple targets interception by the swarm of UAVs based on swarm intelligence algorithms: A review. IETE Tech. Rev..

[32] Tang J., Duan H., Lao S. (2023). Swarm intelligence algorithms for multiple unmanned aerial vehicles collaboration: A comprehensive review. Artif. Intell. Rev..

[33] Aljalaud F., Kurdi H., Youcef-Toumi K. (2023). Bio-inspired multi-UAV path planning heuristics: A review. Mathematics.

[34] Hooshyar M., Huang Y.M. (2023). Meta-heuristic algorithms in UAV path planning optimization: A systematic review (2018–2022). Drones.

[35] Yahia H.S., Mohammed A.S. (2023). Path planning optimization in unmanned aerial vehicles using meta-heuristic algorithms: A systematic review. Environ. Monit. Assess..

[36] Iqbal M.M., Ali Z.A., Khan R., Shafiq M. (2022). Motion planning of UAV swarm: Recent challenges and approaches. Aeronautics-New Advances.

[37] ul Husnain A., Mokhtar N., Mohamed Shah N., Dahari M., Iwahashi M. (2023). A systematic literature review (SLR) on autonomous path planning of unmanned aerial vehicles. Drones.

[38] Agrawal S., Patle B.K., Sanap S. (2024). A systematic review on metaheuristic approaches for autonomous path planning of unmanned aerial vehicles. Drone Syst. Appl..

[39] Debnath D., Vanegas F., Sandino J., Hawary A.F., Gonzalez F. (2024). A review of UAV path-planning algorithms and obstacle avoidance methods for remote sensing applications. Remote Sens..

[40] López B., Mu noz J., Quevedo F., Monje C.A., Garrido S., Moreno L.E. (2021). Path planning and collision risk management strategy for multi-UAV systems in 3D environments. Sensors.

[41] Abujabal N., Fareh R., Sinan S., Baziyad M., Bettayeb M. (2023). A comprehensive review of the latest path planning developments for multi-robot formation systems. Robotica.

[42] Yang Y., Hao J., Lu Z. (2021). A survey of multi-agent reinforcement learning with communication. Neurocomputing.

[43] Javed S., Hassan A., Ahmad R., Ahmed W., Ahmed R., Saadat A., Guizani M. (2024). State-of-the-art and future research challenges in UAV swarms. IEEE Internet Things J..

[44] Mustafa G., Liu Y., Khan I.H., Hussain S., Jiang Y., Liu J., Arshad S., Osman R. (2024). Establishing a Knowledge Structure for Yield Prediction in Cereal Crops Using Unmanned Aerial Vehicles. Front. Plant Sci..

[45] Hu J., Bruno A., Ritchken B., Jackson B., Espinosa M., Delimitrou C., Chae J.Y., Mertil F., Espinosa M., Delimitrou C. (2018). To Centralize or Not to Centralize: A Tale of Swarm Coordination. arXiv.

[46] Arnold R., Mezzacappa E., Jablonski M., Jablonski J., Abruzzo B. (2021). Performance Comparison of Decentralized Undirected Swarms Versus Centralized Directed Swarms at Different Levels of Quality of Knowledge. Proceedings of the 2021 IEEE International Symposium on Technologies for Homeland Security (HST).

[47] Arranz R., Carrami nana D., de Miguel G., Besada J.A., Bernardos A.M. (2025). Application of Deep Reinforcement Learning to UAV Swarming for Ground Surveillance. arXiv.

[48] Qian F., Su K., Liang X., Zhang K. (2023). Task Assignment for UAV Swarm Saturation Attack: A Deep Reinforcement Learning Approach. Electronics.

[49] Hai X., Qiu H., Wen C., Feng Q. (2023). A Novel Distributed Situation Awareness Consensus Approach for UAV Swarm Systems. IEEE Trans. Intell. Transp. Syst..

[50] Cheng Z., Zhao L., Shi Z. (2022). Decentralized Multi-UAV Path Planning Based on Two-Layer Coordinative Framework for Formation Rendezvous. IEEE Access.

[51] Latombe J.C. (1991). Fundamental reference for classical path planning methods like Dijkstra and A*. Robot Motion Planning.

[52] Liu Y., Jebelli H. (2024). Intention-Aware Robot Motion Planning for Safe Worker–Robot Collaboration. Comput. Civ. Infrastruct. Eng..

[53] LaValle S.M. (2006). Covers path planning vs. motion/trajectory planning concepts in detail. Planning Algorithms.

[54] Schöllig A., Mueller M., D’Andrea R. (2012). Trajectory generation for quadrotor swarms. IEEE Trans. Robot..

[55] Zhang Y., Yi P., Hong Y. (2024). Cooperative Safe Trajectory Planning for Quadrotor Swarms. Sensors.

[56] Richter C., Bry A., Roy N. (2016). Polynomial trajectory planning for aggressive quadrotor flight in dense indoor environments. Proceedings of the Robotics Research.

[57] Arshad M.A., Ahmed J., Bang H. (2023). Quadrotor Path Planning and Polynomial Trajectory Generation Using Quadratic Programming for Indoor Environments. Drones.

[58] Xu R., Yao S. (2022). Research on UGV Path Planning in Tunnel Based on the Dijkstra*-PSO* Algorithm. Proceedings of the 2022 6th Asian Conference on Artificial Intelligence Technology (ACAIT).

[59] Reda M., Onsy A., Haikal A.Y., Ghanbari A. (2024). Path Planning Algorithms in the Autonomous Driving System: A Comprehensive Review. Robot. Auton. Syst..

[60] Dorling K., Heinrichs J., Messier G., Magierowski S. (2017). Vehicle routing problems for drone delivery. IEEE Trans. Syst. Man. Cybern. Syst..

[61] Zhang S., Liu S., Xu W., Wang W. (2022). A Novel Multi-Objective Optimization Model for the Vehicle Routing Problem with Drone Delivery and Dynamic Flight Endurance. Comput. Ind. Eng..

[62] Lawler E.L., Lenstra J.K., Rinnooy Kan A.H., Shmoys D.B. (1985). Classic foundational book on TSP algorithms. The Traveling Salesman Problem: A Guided Tour of Combinatorial Optimization.

[63] Marinakis Y. (2024). Heuristic and Metaheuristic Algorithms for the Traveling Salesman Problem. Encyclopedia of Optimization.

[64] Bektas T. (2006). The multiple traveling salesman problem: An overview of formulations and solution procedures. Omega.

[65] Nekovář F., Faigl J., Saska M. (2021). Multi-tour Set Traveling Salesman Problem in Planning Power Transmission Line Inspection. IEEE Robot. Autom. Lett..

[66] Guruji A.K., Agarwal H., Parsediya D.K. (2016). Time-efficient A* algorithm for robot path planning. Procedia Technol..

[67] LaValle S.M. (1998). Rapidly-exploring random trees: A new tool for path planning. Proceedings of the Technical Report TR 98-11.

[68] Xu W., Zhang Y., Yu L., Zhang T., Cheng Z. (2023). A local path planning algorithm based on improved dynamic window approach. J. Intell. Fuzzy Syst..

[69] Wang J., Li Y., Li R., Chen H., Chu K. (2022). Trajectory Planning for UAV Navigation in Dynamic Environments with Matrix Alignment Dijkstra. Soft Comput..

[70] Liu L.S., Lin J.F., Yao J.X., He D.W., Zheng J.S., Huang J., Shi P. (2021). Path Planning for Smart Car Based on Dijkstra Algorithm and Dynamic Window Approach. Wirel. Commun. Mob. Comput..

[71] Du Y. (2023). Multi-UAV Search and Rescue with Enhanced A* Algorithm Path Planning in 3D Environment. Int. J. Aerosp. Eng..

[72] Parkinson J., Patel N. Jump point search enhanced A* for UAV real-time re-planning. Proceedings of the IEEE International Conference on Robotics and Automation (ICRA).

[73] Lee J., Han S. (2022). 3D A* path planning for UAVs using octree-based space partitioning. Robot. Auton. Syst..

[74] Mohamed A., Alsharif K. (2023). Hierarchical A* for large-scale UAV mission planning. Appl. Soft Comput..

[75] Li B., Chen B. (2021). An Adaptive Rapidly-Exploring Random Tree. IEEE/CAA J. Autom. Sin..

[76] Yin H., Li B., Zhu H., Shi L. (2023). Kinodynamic RRT* Based UAV Optimal State Motion Planning with Collision Risk Awareness. Inf. Technol. Control.

[77] Killian L., Backhaus J. (2021). Utilizing the RRT*-Algorithm for Collision Avoidance in UAV Photogrammetry Missions. arXiv.

[78] Chen L. (2021). UAV Path Planning and Obstacle Avoidance Based on Fuzzy Logic and Kinodynamic RRT Methods. Ph.D. Thesis.

[79] Muhsen D.K., Raheem F.A., Sadiq A.T. (2024). Improved Rapidly Exploring Random Tree Using Salp Swarm Algorithm. J. Intell. Syst..

[80] Fox D., Burgard W., Thrun S. (2002). The Dynamic Window Approach to Collision Avoidance. IEEE Robot. Autom. Mag..

[81] Cao Y., Nor N.M. (2024). An Improved Dynamic Window Approach Algorithm for Dynamic Obstacle Avoidance in Mobile Robot Formation. Decis. Anal. J..

[82] Chang X., Chen X., Liu Z., Chen Z., Wang Q., Liu X. (2025). Research on Multi-UAV Autonomous Obstacle Avoidance Algorithm Integrating Improved Dynamic Window Approach and ORCA. Sci. Rep..

[83] Song X., Liu X., Lu J. (2020). Dynamic Local Laplacian Potential Field for UAV Navigation in Unknown Environments. IEEE Trans. Control. Syst. Technol..

[84] Zhang H., Xu S. (2025). Path Planning Technology for Unmanned Aerial Vehicle Swarm Based on Improved Jump Point Algorithm. Int. J. Adv. Comput. Sci. Appl..

[85] Dubins L.E. (1957). On curves of minimal length with a constraint on average curvature, and with prescribed initial and terminal positions and tangents. Am. J. Math..

[86] Moon B., Hong J.H., Mettler E., Rathinam S., Tsiotras P. (2023). Time-Optimal Path Planning in a Constant Wind for Uncrewed Aerial Vehicles Using Dubins Set Classification. IEEE Robot. Autom. Lett..

[87] Wolek A., Seidel J., Kaminer I., Dobrokhodov V., Cobb R., Innes J. (2025). Maximum Kinetic Energy Paths for a Decaying-Speed Dubins Vehicle.

[88] Yan P., Ma L., Li Y., Yu J., Chen C. (2018). A Fixed Wing UAV Path Planning Algorithm Based on Genetic Algorithm and Dubins Curve Theory. Proceedings of the MATEC Web of Conferences.

[89] Wang H., Zhao J. (2023). A novel high-level target navigation pigeon-inspired optimization for global optimization problems. Appl. Intell..

[90] Mirjalili S., Gandomi A.H., Mirjalili S.M., Saremi S., Faris H., Mirjalili S. (2017). Salp Swarm Algorithm: A bio-inspired optimizer for engineering design problems. Adv. Eng. Softw..

[91] Ge F., Wei Y., Yu W., Li J. (2020). Path Planning of UAV for Oilfield Inspections in a Three-Dimensional Dynamic Environment with Moving Obstacles Based on an Improved Pigeon-Inspired Optimization Algorithm. Appl. Intell..

[92] Qiu H., Duan H. (2020). A Multi-Objective Pigeon-Inspired Optimization Approach to UAV Distributed Flocking Among Obstacles. Inf. Sci..

[93] Luo D., Li S., Shao J., Xu Y., Liu Y. (2022). Pigeon-inspired optimisation-based cooperative target searching for multi-UAV in uncertain environment. Int. J. Bio-Inspired Comput..

[94] AlShabi M., Ballous K.A., Nassif A.B., Bettayeb M., Obaideen K., Gadsden S.A. (2024). Path planning for a UGV using Salp Swarm Algorithm. Proceedings of the Autonomous Systems: Sensors, Processing, and Security for Ground, Air, Sea, and Space Vehicles
and Infrastructure 2024.

[95] Singh N., Singh S., Houssein E.H. (2022). Hybridizing Salp Swarm Algorithm with particle swarm optimization algorithm for recent optimization functions. Evol. Intell..

[96] Yao J., Sha Y., Chen Y., Zhang G., Hu X., Bai G., Liu J. (2022). IHSSAO: An improved hybrid Salp Swarm Algorithm and aquila optimizer for UAV path planning in complex terrain. Appl. Sci..

[97] Karaboga D. (2005). An Idea Based on Honey Bee Swarm for Numerical Optimization.

[98] Lin S., Li F., Li X., Jia K., Zhang X. (2022). Improved artificial bee colony algorithm based on multi-strategy synthesis for UAV path planning. IEEE Access.

[99] Sabetghadam B., Cunha R., Pascoal A. (2022). A distributed algorithm for real-time multi-drone collision-free trajectory replanning. Sensors.

[100] Dasgupta A., Zope V., Ismail A. (2025). Implementation of the Bees Algorithm for UAV Mission Plan. Eng. Headw..

[101] Muntasha G., Karna N., Shin S. (2021). Performance analysis on artificial bee colony algorithm for path planning and collision avoidance in swarm unmanned aerial vehicle. Proceedings of the 2021 International Conference on Artificial Intelligence and Mechatronics Systems (AIMS).

[102] Wang R., Shan Y., Sun L., Sun H. (2025). Multi-UAV Cooperative Task Allocation Based on Multi-strategy Clustering Ant Colony Optimization Algorithm. ICCK Trans. Intell. Syst..

[103] Kennedy J., Eberhart R. (1995). Particle Swarm Optimization. Proceedings of the IEEE International Conference on Neural Networks.

[104] Deng L., Chen H., Zhang X., Liu H. (2023). Three-dimensional path planning of UAV based on improved particle swarm optimization. Mathematics.

[105] Yu Z., Si Z., Li X., Wang D., Song H. (2022). A novel hybrid particle swarm optimization algorithm for path planning of UAVs. IEEE Internet Things J..

[106] Yafei W., Liang Z. (2023). Improved multi-objective particle swarm optimization algorithm based on area division with application in multi-uav task assignment. IEEE Access.

[107] Phung M.D., Ha Q.P. (2021). Safety-enhanced UAV path planning with spherical vector-based particle swarm optimization. Appl. Soft Comput..

[108] Bello-Orgaz G., Ramirez-Atencia C., Fradera-Gil J., Camacho D. (2015). GAMPP: Genetic algorithm for UAV mission planning problems. Proceedings of the Intelligent Distributed Computing IX: Proceedings of the 9th International Symposium on Intelligent Distributed Computing–IDC’2015.

[109] Cheng Z., Zhang H., Guo L. (2023). Multi-UAV cooperative task planning based on an improved adaptive simulated annealing and genetic algorithm. Proceedings of the Third International Conference on Advanced Algorithms and Neural Networks (AANN 2023).

[110] Wu X., Yin Y., Xu L., Wu X., Meng F., Zhen R. (2021). Multi-UAV task allocation based on improved genetic algorithm. IEEE Access.

[111] Dharmaraj R., Kumar P., Iqbal M. (2019). Collision-free path planning for UAVs using improved Genetic Algorithms. IEEE Access.

[112] Gyenes Z., Bölöni L., Szádeczky-Kardoss E.G. (2023). Can genetic algorithms be used for real-time obstacle avoidance for lidar-equipped mobile robots?. Sensors.

[113] Mustafa G., Ali Q., Zheng H., Zhou M., Cheng T., Zhu Y., Yao X., Liu Y., Hussain S. (2023). Sensor data fusion and processing in smart agriculture: Crop quality assessment, crop damage, smart planning. Cognitive Sensing Technologies and Applications.

[114] Tong G., Jiang N., Biyue L., Xi Z., Ya W., Wenbo D. (2021). UAV navigation in high dynamic environments: A deep reinforcement learning approach. Chin. J. Aeronaut..

[115] Alam M.M., Trina S.A., Hossain T., Mahmood S., Ahmed M.S., Arafat M.Y. (2025). Variations in Multi-Agent Actor–Critic Frameworks for Joint Optimizations in UAV Swarm Networks: Recent Evolution, Challenges, and Directions. Drones.

[116] Zhang K., Yang Z., Basar T. (2021). Multi-agent reinforcement learning: A selective overview of theories and algorithms. Handbook of Reinforcement Learning and Control.

[117] Zeng F., Wang C., Ge S.S. (2020). A survey on visual navigation for artificial agents with deep reinforcement learning. IEEE Access.

[118] Han L., Zhang H., An N. (2025). A continuous space path planning method for unmanned aerial vehicle based on particle swarm optimization-enhanced deep q-network. Drones.

[119] Osa T., Pajarinen J., Neumann G., Bagnell J.A., Abbeel P., Peters J. (2018). An algorithmic perspective on Imitation Learning. Found. Trends® Robot..

[120] Friston K., FitzGerald T., Rigoli F., Schwartenbeck P., Pezzulo G. (2017). Active inference: A process theory. Neural Comput..

[121] Busoniu L., Babuska R., De Schutter B., Ernst D. (2008). A comprehensive survey of multiagent reinforcement learning. IEEE Trans. Syst. Man. Cybern. Part C (Appl. Rev.).

[122] Hou Y., Zhao J., Zhang R., Cheng X., Yang L. (2023). UAV swarm cooperative target search: A multi-agent reinforcement learning approach. IEEE Trans. Intell. Veh..

[123] De Sá D.F.S., Neto J.V.D.F. (2023). Multi-agent collision avoidance system based on centralization and decentralization control for UAV applications. IEEE Access.

[124] Xia Z., Du J., Wang J., Jiang C., Ren Y., Li G., Han Z. (2021). Multi-agent reinforcement learning aided intelligent UAV swarm for target tracking. IEEE Trans. Veh. Technol..

[125] Arulkumaran K., Deisenroth M.P., Brundage M., Bharath A.A. (2017). Deep reinforcement learning: A brief survey. IEEE Signal Process. Mag..

[126] Zhang W., Zhao L., Liu H. (2022). Multi-UAV path planning based on deep reinforcement learning for MTSP. Aerosp. Sci. Technol..

[127] Watkins C.J., Dayan P. (1992). Q-Learning. Mach. Learn..

[128] Wang S., Qi N., Jiang H., Xiao M., Liu H., Jia L., Zhao D. (2024). Trajectory planning for uav-assisted data collection in iot network: A double deep q network approach. Electronics.

[129] Zhang L., Peng J., Yi W., Lin H., Lei L., Song X. (2023). A state-decomposition DDPG algorithm for UAV autonomous navigation in 3-D complex environments. IEEE Internet Things J..

[130] Konda V.R., Tsitsiklis J.N. (2000). Actor-critic algorithms. Neural Information Processing Systems (NeurIPS).

[131] Grondman I., Busoniu L., Lopes G.A., Babuska R. (2012). A survey of actor-critic reinforcement learning: Standard and natural policy gradients. IEEE Trans. Syst. Man. Cybern..

[132] Gu Y., Cheng Y., Chen C.P., Wang X. (2021). Proximal policy optimization with policy feedback. IEEE Trans. Syst. Man. Cybern. Syst..

[133] Xiang J., Li Q., Dong X., Ren Z. (2019). Continuous control with deep reinforcement learning for mobile robot navigation. Proceedings of the 2019 Chinese Automation Congress (CAC).

[134] Chen Y., Chen R., Huang Y., Xiong Z., Li J. (2024). DRL-Based Improved UAV Swarm Control for Simultaneous Coverage and Tracking with Prior Experience Utilization. Drones.

[135] Huang S., Zhang H., Huang Z. (2022). Multi-UAV Collision Avoidance Using Multi-Agent Reinforcement Learning with Counterfactual Credit Assignment. arXiv.

[136] Hussein A., Gaber M.M., Elyan E., Jayne C. (2017). Imitation Learning: A survey of learning methods. ACM Comput. Surv. (CSUR).

[137] Kim J., Park M., Lee H. (2020). Imitation Learning for UAV swarm formation and coordination. Robot. Auton. Syst..

[138] Wan Y., Tang J., Zhao Z. (2023). Imitation Learning of Complex Behaviors for Multiple Drones with Limited Vision. Drones.

[139] Pan L., Zhou H., Wang Q. (2022). Imitation Learning for multi-UAV cooperative mission planning. IEEE Access.

[140] Zhang L., Xu R., Han Y. (2023). Hybrid imitation–reinforcement learning for UAV swarms in dynamic environments. Appl. Soft Comput..

[141] Friston K., Parr T., Pezzulo G. (2021). The free energy principle: A unified brain theory?. Nat. Rev. Neurosci..

[142] Krayani A., Khan K., Marcenaro L., Marchese M., Regazzoni C. (2023). A Goal-Directed Trajectory Planning Using Active Inference in UAV-Assisted Wireless Networks. Sensors.

[143] Schumann J., Engstroem J., Johnson L., O’Kelly M., Messias J., Kober J., Zgonnikov A. (2025). Active Inference as a Unified Model of Collision Avoidance Behavior in Human Drivers. arXiv.

[144] Smith T., Clark A., Rao V. (2022). Active inference for autonomous UAV navigation in uncertain environments. Neural Netw..

[145] Pezzulo G., Rigoli F., Friston K. (2022). Bayesian Active Inference models for adaptive UAV decision-making. Cogn. Syst. Res..

[146] Millidge B., Tschantz A., Buckley C.L. (2021). Deep Active Inference: Scaling Active Inference using deep learning. Front. Comput. Neurosci..

[147] Arshid K., Krayani A., Marcenaro L., Gomez D.M., Regazzoni C. (2025). UAV Swarm Trajectory Design for Wireless Networks Using Genetic Algorithm-Driven Repulsion Forces. IEEE Access.

[148] Haoran Z., Hang F., Fan Y., Che Q., Yaoming Z. (2024). Data-driven offline reinforcement learning approach for quadrotor’s motion and path planning. Chin. J. Aeronaut..

[149] Zhen Z., Chen Y., Wen L., Han B. (2020). An intelligent cooperative mission planning scheme of UAV swarm in uncertain dynamic environment. Aerosp. Sci. Technol..

[150] Ghdiri O., Jaafar W., Alfattani S., Abderrazak J.B., Yanikomeroglu H. (2021). Offline and online UAV-enabled data collection in time-constrained IoT networks. IEEE Trans. Green Commun. Netw..

[151] Alqefari S., Menai M.E.B. (2025). A Hybrid Method to Solve the Multi-UAV Dynamic Task Assignment Problem. Sensors.

[152] Gopalakrishnan S.K., Al-Rubaye S., Inalhan G. (2021). Adaptive UAV swarm mission planning by temporal difference learning. Proceedings of the 2021 IEEE/AIAA 40th Digital Avionics Systems Conference (DASC).

[153] Rizk Y., Awad M., Tunstel E.W. (2018). Decision making in multiagent systems: A survey. IEEE Trans. Cogn. Dev. Syst..

[154] Zhao L., Chen B., Hu F. (2024). Research on cooperative obstacle avoidance decision making of unmanned aerial vehicle swarms in complex environments under end-edge-cloud collaboration model. Drones.

[155] Sindiramutty S.R. (2025). Swarm intelligence and multi-drone coordination with edge ai. Computer Vision and Edge Computing Technologies for the Drone Industry.

[156] Sönmez S., Rutherford M.J., Valavanis K.P. (2024). A survey of offline-and online-learning-based algorithms for multirotor UAVs. Drones.

[157] Hussain S., Mustafa G., Haider Khan I., Liu J., Chen C., Hu B., Chen M., Ali I., Liu Y. (2023). Global trends and future directions in agricultural remote sensing for wheat scab detection: Insights from a bibliometric analysis. Remote Sens..

[158] Rezaee M.R., Hamid N.A.W.A., Hussin M., Zukarnain Z.A. (2024). Comprehensive review of drones collision avoidance schemes: Challenges and open issues. IEEE Trans. Intell. Transp. Syst..

[159] Singh A., Payal A. Development of a Low-Cost Collision Avoidance System Based on Coulomb’s Inverse-Square Law for Multi-Rotor Drones (UAVs). Proceedings of the 2021 International Conference on Computational Performance Evaluation (ComPE).

[160] Salve S.S., Chaudhari S.Y., Dandekar A.R., Gaikwad P. (2025). Anti Collision Drone Traffic Control System Using Swarm Technology. https://www.researchgate.net/publication/393297933_Anti_Collision_Drone_Traffic_Control_System_Using_Swarm_Technology.

[161] Liu W., Zhang B., Liu P., Pan J., Chen S. (2024). Velocity obstacle guided motion planning method in dynamic environments. J. King Saud Univ.-Comput. Inf. Sci..

[162] Liu W.h., Zheng X., Deng Z.h. (2021). Dynamic collision avoidance for cooperative fixed-wing UAV swarm based on normalized artificial potential field optimization. J. Cent. South Univ..

[163] Dang A.D., La H.M., Nguyen T., Horn J. (2019). Formation control for autonomous robots with collision and obstacle avoidance using a rotational and repulsive force–based approach. Int. J. Adv. Robot. Syst..

[164] Liu J., Wang Y., Huang P.Q., Jiang S. (2021). Car: A cutting and repulsion-based evolutionary framework for mixed-integer programming problems. IEEE Trans. Cybern..

[165] Ajith V., Jolly K. (2023). Hybrid optimization based multi-objective path planning framework for unmanned aerial vehicles. Cybern. Syst..

[166] Mozga N., Gutans J., Kubulins R., Chatys R. (2024). Calculation and design of the main equipment for mobile space simulation system. Trans. Aerosp. Res..

[167] Xu P., Liu J., Sun X., Chen H., Chen Y. (2024). Distributed Consensus Control Research of Unmanned Aerial Vehicle (UAV) Swarms Based on Lennard–Jones Potential. Proceedings of the International Conference on Machine Learning, Cloud Computing and Intelligent Mining.

[168] Masoud A.A. (2012). A harmonic potential approach for simultaneous planning and control of a generic UAV platform. J. Intell. Robot. Syst..

[169] Mok J., Lee Y., Ko S., Choi I., Choi H.S. (2017). Gaussian-mixture based potential field approach for UAV collision avoidance. Proceedings of the 2017 56th Annual Conference of the Society of Instrument and Control Engineers of Japan (SICE).

[170] Tang H., Dou H., Gao Q., Mao Z., Ji Y., Liu J. (2025). An Improved Gaussian Sampling-Based Bidirectional RRT Algorithm in 3D Path Planning for Low-Altitude Urban Environments. Proceedings of the 2025 37th Chinese Control and Decision Conference (CCDC).

[171] Khatib O. (1986). Real-time obstacle avoidance for manipulators and mobile robots. Int. J. Robot. Res..

[172] Balanji H.M., Yanmaz E. (2024). Priority-based dynamic multi-UAV positioning for multi-target search and connectivity. Proceedings of the 2024 IEEE Wireless Communications and Networking Conference (WCNC).

[173] Iqbal H., Sadia H., Al-Kaff A., Garcia F. (2025). Novelty Detection in Autonomous Driving: A Generative Multi-Modal Sensor Fusion Approach. IEEE Open J. Intell. Transp. Syst..

